# Insights into the Genome of Large Sulfur Bacteria Revealed by Analysis of Single Filaments

**DOI:** 10.1371/journal.pbio.0050230

**Published:** 2007-08-28

**Authors:** Marc Mußmann, Fen Z Hu, Michael Richter, Dirk de Beer, André Preisler, Bo B Jørgensen, Marcel Huntemann, Frank Oliver Glöckner, Rudolf Amann, Werner J. H Koopman, Roger S Lasken, Benjamin Janto, Justin Hogg, Paul Stoodley, Robert Boissy, Garth D Ehrlich

**Affiliations:** 1 Max Planck Institute for Marine Microbiology, Bremen, Germany; 2 Center for Genomic Sciences, Allegheny General Hospital/Allegheny-Singer Research Institute, Pittsburgh, Pennsylvania, United States of America; 3 School of Engineering and Sciences, Jacobs University Bremen, Bremen, Germany; 4 Department of Membrane Biochemistry, Nijmegen Centre for Molecular Life Sciences, Radboud University Nijmegen Medical Centre, Nijmegen, The Netherlands; 5 J. Craig Venter Institute, Rockville, Maryland, United States of America; University of Arizona, United States of America

## Abstract

Marine sediments are frequently covered by mats of the filamentous *Beggiatoa* and other large nitrate-storing bacteria that oxidize hydrogen sulfide using either oxygen or nitrate, which they store in intracellular vacuoles. Despite their conspicuous metabolic properties and their biogeochemical importance, little is known about their genetic repertoire because of the lack of pure cultures. Here, we present a unique approach to access the genome of single filaments of *Beggiatoa* by combining whole genome amplification, pyrosequencing, and optical genome mapping. Sequence assemblies were incomplete and yielded average contig sizes of approximately 1 kb. Pathways for sulfur oxidation, nitrate and oxygen respiration, and CO_2_ fixation confirm the chemolithoautotrophic physiology of *Beggiatoa*. In addition, *Beggiatoa* potentially utilize inorganic sulfur compounds and dimethyl sulfoxide as electron acceptors. We propose a mechanism of vacuolar nitrate accumulation that is linked to proton translocation by vacuolar-type ATPases. Comparative genomics indicates substantial horizontal gene transfer of storage, metabolic, and gliding capabilities between *Beggiatoa* and cyanobacteria. These capabilities enable *Beggiatoa* to overcome non-overlapping availabilities of electron donors and acceptors while gliding between oxic and sulfidic zones. The first look into the genome of these filamentous sulfur-oxidizing bacteria substantially deepens the understanding of their evolution and their contribution to sulfur and nitrogen cycling in marine sediments.

## Author Summary

In 1888 Winogradsky proposed the concept of chemolithotrophy—growth using inorganic compounds as an energy source—after studying the sulfur bacterium *Beggiatoa*. These filamentous bacteria and related organisms inhabit the surface of marine and freshwater sediments, where they oxidize hydrogen sulfide using either oxygen or nitrate. In particular, conspicuously large marine representatives accumulate nitrate in vacuoles to survive anoxia, a unique feature among prokaryotes. Since nitrate-storing *Beggiatoa* are not available in pure culture, we amplified and sequenced the genomic DNA of single multicellular filaments. We comprehensively tested the incomplete sequence assemblies for foreign DNA. We show that the *Beggiatoa* genome encodes the pathways of chemolithoautotrophy but also appears to support the use of alternative electron donors and acceptors. We propose that vacuolar-type ATPases generate an electrochemical gradient to drive nitrate transport over the vacuole membrane, a mechanism similar to eukaryotic solute accumulation. Intriguingly, we found evidence for substantial gene exchange between *Beggiatoa* and cyanobacteria. In both phyla, hemagglutinins are possibly involved in filament formation. The breadth of storage and metabolic capabilities encoded in its genome enables *Beggiatoa* to act as a “rechargeable battery,” which glides between oxic and sulfidic zones to overcome non-overlapping availabilities of electron donors and acceptors.

## Abstract 3

Examining the genome of single filaments of *Beggiatoa* deepens our understanding of their contribution to sulfur and nitrogen cycling in marine sediments.

## Introduction

Mats of conspicuously large sulfur-oxidizing bacteria often cover the seafloor in organicly rich coastal areas, at hydrate ridge methane seeps, at hydrothermal vents, on whale falls, and in coastal upwelling regions [1–5]. The closely related genera *Beggiatoa, Thioploca,* and *Thiomargarita* are among the largest prokaryotes known, and they usually contain a vacuole that can account for up to 90% of the cell volume [6]. On the seafloor these large sulfur-oxidizing bacteria fulfill an important ecological function by preventing the release of toxic hydrogen sulfide from the sediment into the water column. Studying *Beggiatoa,* Winogradsky [7] demonstrated the principle of chemolithotrophy, a process in which the oxidation of inorganic sulfur is coupled to oxygen respiration. By their gliding motility *Beggiatoa* aggregate at the oxic–anoxic transition zone, where oxygen and sulfide occur in opposed diffusion gradients [3,8]. *Beggiatoa* compete using chemical sulfide oxidation [8,9], mainly by Fe(III), and can significantly contribute to biological sulfur oxidation [10,11]. Oxygen has been regarded as the major electron acceptor coupled to sulfur oxidation; however, there is growing evidence that when experiencing anoxia these large vacuolated *Beggiatoa, Thioploca,* and *Thiomargarita* respire nitrate, which they concentrate up to 10,000-fold (∼500 mM) within their intracellular vacuoles [5,12,13]. Their nitrate and sulfur storage capacities allow them to bridge the suboxic zone, where neither sulfide nor oxygen is detectable, which gives them an advantage over other sulfide-oxidizing bacteria. In addition, these large sulfur-oxidizing bacteria may release phosphate from accumulated polyphosphate (polyP), which has been hypothesized to account for the large phosphorite deposits on the seafloor [14,15].

None of these large nitrate-storing bacteria are available in pure culture. Thus, little is known about the gene content associated with their chemolithotrophic properties, their conspicuous morphology, or their exceptional nitrate storage abilities. Previous physiological and genetic studies were mainly performed on the small, readily culturable, non-vacuolated *B. alba,* a species that is phylogenetically distant from the large sulfur-oxidizing bacteria [16]. Because of phenotypic similarities such as gliding motility and filamentous shape, Beggiatoa spp. were regarded as colorless cyanobacteria (discussed in [17]) before they were reclassified as Gammaproteobacteria based on 16S rRNA gene sequences.

It is now standard to study large genomic fragments of uncultured microbes by shotgun cloning and sequencing of bulk DNA extracted from mixed communities [18–20]; however, assembly of genomes for discrete species is problematic. Alternatively, DNA can be exponentially amplified (up to 10^9^-fold) from single cells [21] by multiple displacement amplification (MDA) [22–25], enabling sequencing from uncultured microorganisms isolated from the environment [25–27]. Despite background amplification and chimera formation [28], this method amplifies complex DNA much more faithfully than earlier whole genome amplification (WGA) strategies. Recently, more than 60% of the genome of single cultured *Prochlorococcus* cells were amplified and sequenced with improved methods that greatly reduced background amplification and chimera formation [29]. Here, the cloning of amplified, hyperbranched DNA was suspected to facilitate the formation of chimeric sequences. However, chimeric sequences can occur to a similar extent in pyrosequenced datasets [28], indicating that MDA is the causative agent in chimera formation. Non-electrophoretic sequencing methods such as pyrosequencing [30] offer the advantage of massively parallel sequencing of large numbers of DNA fragments without cloning and hence less chimera formation. They also obviate the problems of cloning bias and of sequencing GC-rich DNA.

The combination of the low representational bias of MDA-amplified genomic DNA with the advantages of clone-free pyrosequencing augers well for the great potential to rapidly analyze the genomes of unculturable microbes. Here, we report what is to our knowledge the first large-scale genomic analysis of an uncultured, environmental bacterium based on WGA and pyrosequencing. Using MDA the genomic DNA of two individual multicellular (>600 cells) filaments of uncultured *Beggiatoa* (∼30 μm in diameter) from a Baltic Sea harbor sediment were separately amplified. One of these amplification products was sequenced using a clone-free pyrosequencing method developed by 454 Life Sciences [31]; the other was sequenced using electrophoretic (Sanger) sequencing of clone libraries. To estimate the heterogeneity among individual *Beggiatoa* filaments and the proportion of the *Beggiatoa* genome covered by our sequences, the genome size was independently determined by optical mapping [32] using filaments of co-occurring *Beggiatoa*.

## Results/Discussion

### *Beggiatoa* as a Gradient Organism

Here, we present the draft genome sequences of two individual filaments of Beggiatoa sp. recovered from the surface of a marine sediment. The sediment–water interface in marine and freshwater habitats is characterized by steep gradients of electron donors and acceptors such as sulfide, oxygen, and nitrate. Since the zones of availabilities of electron donors and acceptors usually do not overlap, nitrate-storing *Beggiatoa* move between the oxic and sulfidic sediment layers to overcome this limitation. In the following, the general genome features and genome-encoded adaptations for this lifestyle in two individual *Beggiatoa* filaments are illustrated. In particular, we focus on the chemolithotrophy and the unique storage capabilities of the vacuolated *Beggiatoa.* Furthermore, we provide evidence of horizontal gene transfer with cyanobacteria, which likely reflects the long-term coexistence of these two phyla at sediment surfaces.

### Optical Mapping and Genome Size Estimation Using Filaments of Uncultured *Beggiatoa*

**Figure 1:**
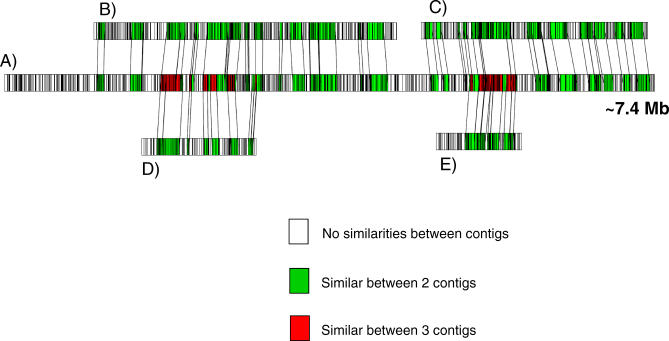
Optical Mapping of the Genomes of Five Pooled *Beggiatoa* Filaments (35 μm in Diameter) Contigs after assembly of restriction patterns using the enzyme AflII. (A) shows the circular chromosome of 7.4 Mb; (B–D) display linear contigs. The lines connect regions of significantly similar restriction patterns. Green indicates regions displaying similarity between two contigs; red indicates regions displaying similarity between three contigs; white indicates no similarities between contigs.

**Figure 2:**
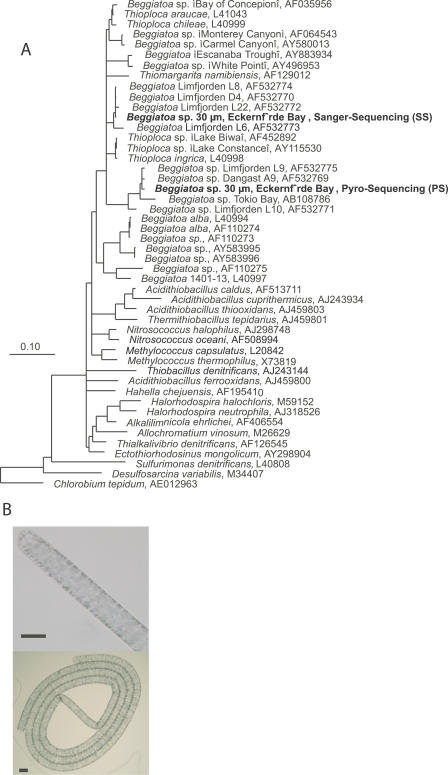
*Beggiatoa* Sp. Phylogeny and Morphology (A) Phylogenetic reconstruction based on the 16S rRNA genes encoded on the PS and SS datasets. Branching orders that were not supported by all methods are shown as multifurcations. Partial sequences were subsequently inserted into the reconstructed consensus tree. The scale bar corresponds to 10% estimated sequence divergence. (B) Micrographs of two multicellular filaments of vacuolated *Beggiatoa* from Eckernförde Bay/Baltic Sea. Scale bars correspond to 30 μm.

Comprehensive genomic analysis of specific environmental microorganisms is hampered by a high microdiversity of co-occurring and closely related organisms [33]. Hence, accurate estimates of sequence heterogeneity and genome size are required. To estimate the heterogeneity and the genome size of large, uncultured *Beggiatoa,* we performed optical mapping of single DNA molecules. Unamplified, high-molecular-weight DNA molecules were isolated from five co-occurring, 35-μm-diameter filaments, each composed of more than 600 putatively clonal cells descended from the filament's progenitor cell. We used a small number of morphologically identical *Beggiatoa* filaments to reduce the risk of obtaining mapping data compromised by co-occurring and closely related organisms. The DNA from the *Beggiatoa* yielded a consensus optical map of a single circular chromosome of approximately 7.4 Mb (Figures 1A and S1). This is over twice the estimated size (3 Mb) of the genome of the non-vacuolated species B. alba [34]. Consensus maps were also obtained for four linear contigs, with sizes ranging from 0.9 to 3.4 Mb (Figure 1B–1E). In some regions the restriction patterns of the consensus maps of these smaller linear contigs were similar to regions of the consensus map of the larger circular chromosome, whereas other regions were highly dissimilar. The diverging DNA restriction patterns of the five contig maps are likely not attributable to an unusually high genome plasticity but rather reflect the high microdiversity among the five *Beggiatoa* filaments, as has been reported for marine Vibrio spp. [33]. This led us to sequence the genome of a single filament rather than the metagenome of a mixed community of closely related species (Figure 2B).

### Specificity of WGA

WGA using MDA from a single or a few cells is highly sensitive to random DNA synthesis. It is also compromised by the presence of non-target DNA, which is a major concern particularly in environmental projects. To minimize these problems we obtained *Beggiatoa* DNA from a well-purified multicellular filament consisting of more than 600 cells to provide a large number of putatively clonal chromosome copies as a template for WGA. Consistently, the data analysis strongly suggests successful amplification and assembly of genomic DNA from *Beggiatoa* filaments cells exclusively, even though the filaments had been obtained directly (without prior cultivation) from a marine sediment.

### DNA Sequencing and General Genome Features

The whole genomic DNA of a single filament was amplified using MDA. From the amplified DNA a clone library was constructed that was Sanger sequenced (SS dataset). This approach yielded a low-coverage (3×) partial assembly of 1,091 contigs with a total length of 1.3 Mb (Table 1). In a separate experiment the DNA from a second filament was amplified and subsequently pyrosequenced (PS dataset). The PS assembly achieved a high coverage depth (17×) and a total length of 7.6 Mb. A detailed overview of the sequencing results and preliminary genome features are given in Tables 1, S1, and S2. The maximum contig size was 18.6 kb for the PS genome and 5.5 kb in the SS genome.

**Table 1:**
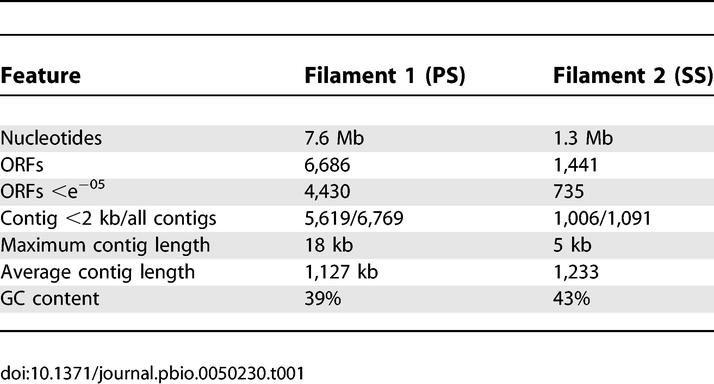
Comparison of General Features of the *Beggiatoa* Genome Sequence Assemblies after WGA and Two Distinct Sequencing Methods

For open reading frame (ORF) prediction, only contigs larger than 2 kb were considered. The average ORF length was 594 bp (SS2) and 827 bp (PS2). The high number of short, non-overlapping contigs (Table 1) suggests a genome larger than 7.6 Mb. Reconciliation of the optical genome map of *Beggiatoa* (Figures 1 and S1) with the *Beggiatoa* PS genome sequence was impractical because of the incomplete sequence assembly.

The low level of sequence assembly is not attributed to high genome plasticity among cells in a single filament. We assume that a multicellular filament is derived from one progenitor cell and thus is clonal. It is highly unlikely that massive genome rearrangements occur within approximately ten generations (2^9^–2^10^ cell divisions = 512–1,024 cells/filament). Thus, the sequence dataset of each filament represents the genome of a single strain rather than a population of slightly different genomes or even a metagenome of mixed organisms.

Several tests at different stages of this study were conducted to determine if there was any significant contribution of potential non-*Beggiatoa* DNA to the PS sequence assembly: (1) an analysis of the PS sequence read metadata; (2) an analysis of intrinsic DNA signatures of the assembled sequences, and (3) genome annotation and phylogenetic reconstruction of different marker molecules and analysis of single-copy genes. The results of these analyses are highly consistent with the claim that the assembled sequences are derived from *Beggiatoa* only.

### Repeat and Singleton PS Reads

Reads from repeat regions (excluded from an assembly) were an unusually high percentage (11.3%) of the PS reads (Tables S1 and S3). It is unclear if this reflects the repetitive DNA content of the *Beggiatoa* genome, or if this is an artifact of WGA. The assembled and repeat reads had similar properties (Table S2), and there were multiple examples of repeat reads with more than ten copies, indicating they were not randomly amplified DNA. These data provide a possible explanation for the large number of contigs in the PS assembly, as repeat reads typically result in gaps that an assembler cannot resolve.

In addition, approximately 10 Mb of PS reads were singletons (5.1% of the total reads), which had a significantly different GC content (42.5%) than the assembled reads (Tables S1 and S2). Singleton reads may originate from randomly amplified DNA, from *Beggiatoa* DNA sequences that amplify poorly, or from non-*Beggiatoa* DNA. They could represent a random sampling of trace contaminating DNA that has a potentially very large complexity but very low copy number per discrete contaminating genome. Although contamination cannot be completely ruled out, there was probably not enough non-*Beggiatoa* DNA present in the MDA reaction to yield sufficient read coverage depth to significantly affect the sequence assembly. Moreover, our analyses of nucleotide composition, single-copy genes, and 16S rRNA genes (see below) also do not support significant contribution of non-*Beggiatoa* DNA.

### Nucleotide Composition and Binning of Sequences

To identify potentially contaminating DNA sequences in our assembled data, all contigs of the PS dataset (7.6 Mb) were analyzed in a binning approach based on intrinsic DNA signatures. Relative abundance of dinucleotides, Markov-model-based statistical evaluations of tri- and tetramer over- and underrepresentation, and normalized chaos game representations for tri- and tetramers were investigated. This approach has been shown to enable a highly sensitive clustering of DNA sequences even among closely related gammaproteobacteria [35]. In the *Beggiatoa* PS dataset no outliers were identified that would indicate potentially contaminating DNA (data not shown).

### 16S rRNA Phylogeny

*Beggiatoa* is a representative of the large sulfur-oxidizing bacteria that form a monophyletic cluster within the Gammaproteobacteria [36]. In both genomes we identified partial 16S rRNA gene sequences that were highly similar to sequences of marine *Beggiatoa* (Figure 2A). The gammaproteobacterial affiliation is supported by the phylogeny of a set of 41 concatenated proteins (Figure S2). Comparative sequence analysis revealed that the two *Beggiatoa* filaments (PS and SS datasets) are phylogenetically different despite their similar diameter of approximately 30 μm. This result is consistent with the potential genomic microdiversity among filaments indicated by the optical mapping results. The distinct phylogenetic origin is also reflected in the GC contents of both sequence datasets, which differ by 4% (Table 1). No additional 16S rRNA gene sequences were found.

### Phylogenetic Affiliation of Genes

Based on 16S rRNA sequence similarity, Nitrosococcus oceani and Methylococcus capsulatus are the closest relatives of *Beggiatoa* for which whole genome sequences are available. An analysis of the conserved ORFs for best BLAST hits against a local genome database was largely consistent with this affiliation (Table S3).

As both filaments are closely related at the 16S rRNA gene level, a large fraction of genes in both datasets were expected to be likewise highly similar. Therefore, the ORFs of the PS2 and SS2 datasets were compared for reciprocal best match (RBM) hits. In both datasets 378 ORFs mutually display the highest similarity (cut-off of e^−05^, 65% minimum sequence coverage). Because of only partially covered genes, many ORFs present in both genomes were not apparent despite showing their highest sequence similarities to the other sequenced *Beggiatoa* genome after manual reinvestigation. Thus, the observed number of ORFs with RBM hits constitutes only the minimum.

Interestingly, many ORFs showed their highest similarity to genes from the filamentous Nostoc sp. and gliding Anabaena variabilis. Furthermore, some gene fragments are exclusively shared with cyanobacteria, among them Nostoc sp., *Gloeobacter violaceus,* and A. variabilis. Most of these ORFs encode conserved hypothetical genes, of which many show similarities to putative transposases (e.g., BgP0160 and BgP1020ff), reverse transcriptase, and fdxN element excision controlling factor proteins. ORF BgP4037 encodes a conserved hypothetical protein (196 aa) with the highest sequence similarity (58%) to predicted proteins of Trichodesmium sp. (Figure S3A), of which at least 30 paralogs are present in the PS dataset. Moreover, BgP4037 co-localizes with “authentic” *Beggiatoa* genes such as nitrate reductase subunit genes (Figure S3B). The phylogenetic reconstructions of proteins containing either adenylation domains (AMP-A) or hemagglutinin domains (Figures S3 and S4; see below) confirm the hypothesis of horizontal gene transfer. Furthermore, contigs carrying cyanobacterial-like genes did not group in the cluster analysis, which indicates an already *Beggiatoa*-adapted codon usage pattern. In conclusion, these findings suggest extensive gene exchange between (filamentous) cyanobacteria and *Beggiatoa*. This apparent gene sharing is particularly interesting since *Beggiatoa* was formerly classified as a colorless cyanobacterium because of many shared phenotypic characteristics (for review see [17]).

### Ribosomal Proteins, Amino-Acyl tRNA Synthetases and Single-Copy Genes

To estimate the extent of putative contaminating DNA, in particular of cyanobacterial origin, we searched for duplicate genes that usually occur only once per prokaryotic genome. We identified 47 ribosomal proteins in the PS dataset that exclusively affiliated with Gammaproteobacteria (Table S4)*.* The gammaproteobacterial affiliation is well confirmed by the phylogenetic reconstruction of a set of 41 concatenated proteins comprising 39 ribosomal proteins, recombinase A (recA), and RNA polymerase subunit B (Figure S2). Recently, a novel approach for the prediction of the number of genome equivalents in metagenomic samples was proposed [37] that is based on the occurrence of 35 widely conserved, single-copy marker genes present in most prokaryotic genomes. Out of these 35 we identified 30 genes (Table S5) in the PS dataset, none of which were found more than once. In addition, we found 40 genes of an extended set of 55 single-copy genes that are not as widely distributed (Table S6). Consistent with these findings 18 out of 24 amino-acyl tRNA synthetase genes were observed as single-copy genes in the PS dataset (Table S7). In conclusion, the single occurrence of proposed single-copy genes, ribosomal proteins, and amino-acyl tRNA synthetases is indicative of the presence of a single dominant genome in the assembled DNA sequence. Alternative phylogenetic markers such as recA, ATP synthase subunits, elongation factor Tu, RNA polymerase, and DNA gyrase AB were most similar to the Gammaproteobacteria based on BLASTP analysis. The only exception was a heat shock protein, Hsp70 *(dnaK),* that affiliated with Hsp70 of *Firmicutes.* However, it is known that Hsp70 genes are horizontally exchanged [38,39].

The genome size of *Beggiatoa* was estimated based on the ratio of single-copy marker genes, amino-acyl tRNA synthetase genes, and tRNA genes to their expected values. This suggests a genome coverage of more than 70% by the PS data, or a genome size of up to 11 Mb.

### Sulfur Oxidation

In 1888 Winogradsky [7] demonstrated the concept of chemolithotrophy studying a freshwater *Beggiatoa*. He showed that *Beggiatoa* gain electrons from oxidization of hydrogen sulfide to elemental, intracellularly stored sulfur and further to sulfate. However, the detailed pathways of sulfur species oxidation in these bacteria have not been elucidated.

**Figure 3:**
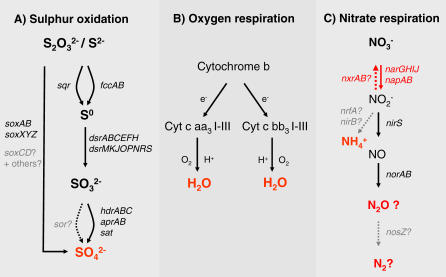
Predicted Sulfur, Oxygen, and Nitrogen Metabolism of *Beggiatoa* Sp. (A) Overview of the encoded genes catalyzing sulfur species oxidation. A sulfite acceptor oxidoreductase was not indicated, in contrast to earlier experimental evidence in non-vacuolated *Beggiatoa* [47]. Note that thiosulfate is probably oxidized via the Sox pathway. (B) Final steps in oxygen respiration. The depicted cytochrome c oxidases show different affinities to oxygen: the cbb_3_ type has a higher affinity than the aa_3_ type. (C) Nitrate respiration. Enzymes reducing nitrite to ammonia and nitrous oxide to dinitrogen, respectively, were not found.

Recent studies on nitrate-respiring *Beggiatoa* pointed to a two-step oxidation of sulfide [11,40]. In the anoxic zone sulfide is oxidized to elemental sulfur and sulfate at the expense of (stored) nitrate. Then *Beggiatoa* moves upwards into the oxic zone, where the stored elemental sulfur is further oxidized to sulfate using oxygen. When shuttling between sediment layers *Beggiatoa* experiences variable sulfide concentrations [41]. The initial oxidation of hydrogen sulfide to elemental sulfur is probably catalyzed via either of two alternative pathways: (1) a sulfide quinone oxidoreductase (Sqr) or (2) a flavocytochrome c/sulfide dehydrogenase (FccAB) (Figure 3A). Sqr is widespread among prokaryotes and appears to be critical for sulfide oxidation in Allochromatium vinosum [42]. FccAB was hypothesized to be more prevalent at low sulfide concentrations [43] and may be more important in the upper, oxidized sediment layers.

The genomes of both *Beggiatoa* filaments encode proteins of the “reverse dissimilatory sulfate reductase (rDsr) pathway” [44,45] (Figure 3A). We identified gene fragments encoding the cytoplasmic rDsrABC and also the membrane proteins DsrMKJOP that channel electrons to rDsrAB. Similar to in the betaproteobacterium Thiobacillus denitrificans [46], at least five paralogs of the DsrC-like subunit are present in the *Beggiatoa* genome (PS2). After formation of sulfite by DsrABC, it is oxidized and phosphorylized by an adenosin-phosphosulfate (APS) reductase to APS [47]. Finally, APS is dephosphorylized via an ATP sulfurylase to yield sulfate and ATP [47]. In *Beggiatoa* the AprAB is functionally linked to heterodisulfide reductases (HdrABC) that are likely responsible for electron transport to AprAB, as suggested for sulfate-reducing prokaryotes [48,49].

In *Beggiatoa* the oxidation of thiosulfate is catalyzed by the identified SoxABXYZ subunits of the Sox pathway [50]. However, so far all investigated organisms encoding the rDsr pathway lack the Sox(C)D subunits [51]. Simultaneously these organisms form sulfur globules while oxidizing reduced sulfur compounds. This is consistent with the observed sulfur globule formation and the missing SoxCD genes in *Beggiatoa,* but their presence in the unsequenced part of the genome cannot be excluded yet. In these organisms and most likely also in *Beggiatoa* rDsrAB is crucially involved in further oxidizing transiently stored elemental sulfur to sulfite [52]. Thus, the rDsr pathway is likely essential for *Beggiatoa* to perform an energetically more favorable two-step oxidation of sulfide and sulfur using nitrate and oxygen, respectively [11], when the zones of oxygen and sulfide do not overlap.

### Oxygen Respiration

In organic-rich surface sediments oxygen is rapidly consumed. In typical *Beggiatoa* habitats oxygen penetrates only the upper few millimeters. Culturable *Beggiatoa* and their relatives commonly exhibit a negative chemotactic response to high oxygen concentrations [53], and preferentially oxidize inorganic sulfur compounds under microoxic conditions. The presence of high- and low-affinity terminal oxidases in both *Beggiatoa* datasets reflects the flexibility to respond to different oxygen regimes (Figure 3B). Under high oxygen concentrations a low-affinity cytochrome c aa_3_-oxidase is predicted to be used, whereas under microoxic conditions a high-affinity cytochrome c bb_3_-oxidase may be more prevalent. The differential expression of cytochrome oxidases under oxic and microoxic conditions has been reported for the freshwater relative B. leptomitiformis [54].

### Nitrate Respiration

Vacuolated marine *Beggiatoa* and their relatives most likely respire nitrate under anoxic conditions [11,12,55]. The PS dataset encodes both membrane-bound (NarGH) and periplasmic (NapAB) nitrate reductases (Figure 3C). Because of the incomplete assembly, three non-overlapping fragments of a NarG gene were found (BgP3372, BgP5024, and sequences downstream of BgP4047) that were concatenated and phylogenetically affiliated with Proteobacteria (Figure S6). In addition to these proteobacterial NarGH, we surprisingly identified a second nitrate reductase, NarGH (BgP0139 and BgP4784), displaying by far the highest sequence similarities (NarG: 57% similarity at 98% coverage) to a putative nitrate reductase/nitrite oxidoreductase of the anaerobically ammonia-oxidizing planctomycete Kuenenia stuttgartiensis [56]. The phylogenetic reconstruction of both sequences revealed a novel lineage of putative nitrate reductases (Figure S6). However, nitrate reductases can also operate in the reverse direction in nitrite-oxidizing bacteria, where they are considered nitrite oxidoreductases (Nxr) [57]. Since there is physiological evidence for nitrite oxidation in K. stuttgartiensis with the NarG as candidate enzyme (M. Strous, personal communication), we speculate that *Beggiatoa* also utilize nitrite as an electron donor. In general, the function of NapAB (BgP1197ff) is unclear, but it may allow *Beggiatoa* to support nitrate respiration at low nitrate concentrations [58] or may enable *Beggiatoa* to respire nitrate even under aerobic conditions [59].

The preferred pathway of nitrate respiration in *Beggiatoa* and relatives and its regulations are of major ecological importance [60]. It is assumed that the main product of nitrate respiration in marine *Beggiatoa* and relatives is ammonia [16]. Although we could not identify the enzymes catalyzing the final reduction steps to ammonium ion or molecular nitrogen, they may be encoded on the not-yet-sequenced part of the genome. In *Beggiatoa,* a nitrite reductase (*nirS;* BgP1272) and two nitric oxide reductases (*norB;* BgP5178 and BgP3622) reduce nitrite and nitric oxide, respectively, to nitrous oxide (Figure 3C). To experimentally test the capability of *Beggiatoa* to denitrify, we measured nitrous oxide formation in acetylene-inhibited natural mats of nitrate-storing *Beggiatoa* in arctic marine sediments. The natural mat of *Beggiatoa* dissimilatorily reduced nitrate to nitrous oxide, while the adhering *Beggiatoa*-free sediment did not (Figure S7). In summary, the genomic and experimental data presented here provide a first clear indication of the significant denitrification potential of large marine sulfur bacteria.

### Vacuolar Storage of Nitrate

**Figure 4:**
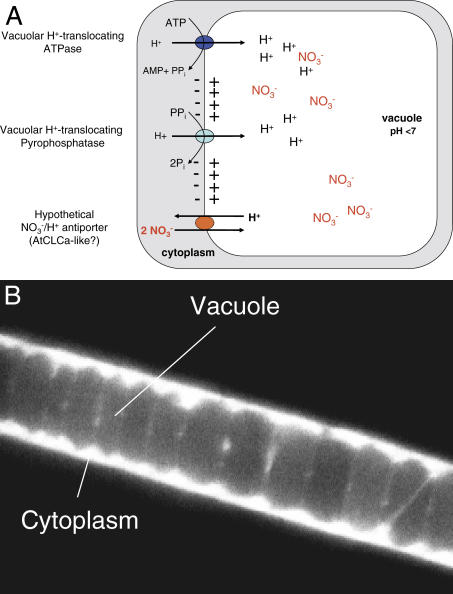
Energetic Vacuolar Concentration of Nitrate by *Beggiatoa* Sp. (A) Hypothetical model of nitrate accumulation in the vacuole of *Beggiatoa.* (B) *Beggiatoa* filament stained with the cationic, lipophilic dye rhodamine 123. Rhodamine 123 accumulated in the cytoplasm but was excluded from vacuoles, indicating the presence of an electric potential (inside positive) over this membrane.

The large, vacuolated *Beggiatoa* and relatives are unique among prokaryotes in their exceptional nitrate storage capabilities. They accumulate nitrate internally to high concentrations of up to 500 mM [16], which allows them to monopolize nitrate and therefore to outcompete other denitrifying bacteria [11]. The underlying physiological and genetic mechanisms of nitrate accumulation are still unknown. Plants store up to 50 mM nitrate in their vacuoles [61]. Here, the uptake of nitrate across the cytoplasmic membrane is usually driven by a transmembrane electrochemical gradient (Δp) followed by a transport of nitrate [62]. In plants, typically vacuolar-type H^+^-ATPases and H^+^-pyrophosphatases (HPPases) catalyze a proton translocation over endomembranes to generate a Δp for solute transport and likely also nitrate transport [63]. Vacuolar-type ATPases also occur in plasma membranes of some Archaea, but they are rarely encountered in Bacteria [64,65]. We propose that the accumulation of nitrate in *Beggiatoa* may be driven by a ΔpH generated by vacuolar-type ATPases and PPases. This energy is used by probable H^+^/Cl^−^ exchanger-like proteins to exchange the accumulated protons in the vacuole and nitrate in the cytoplasm (Figure 4A). In support of this hypothesis we identified six of the nine putative subunits of vacuolar-type H^+^/Na^+^-translocating ATPase (*atpABCDEI*) (Figure 4A), which show their highest similarity to homologs in *Nitrosococcus oceani,* a related organism also containing intracellular membrane vesicles. Furthermore, a vacuolar H^+^-pyrophophatase *(hppA)* and an uncommon Ca^2+^-translocating ATPase were identified in the PS dataset that may also contribute to generation of a Δp/ΔpH (Figure 4A). To check for the presence of an electric potential (inside positive) over the vacuolar membrane, filaments were stained with fluorescent lipophilic cation rhodamine 123. The fact that rhodamine 123 was excluded from the vacuole of *Beggiatoa* cells is consistent with our hypothesis (Figure 4B). Considering the presumed ΔpH and the measured high nitrate concentrations in *Beggiatoa,* a corresponding acidic pH of the vacuole content similar to that observed in plants [66,67] would be predicted. In fact, preliminary pH measurements of the vacuole content of Beggiatoa sp. and Thiomargarita namibiensis (data not shown) give additional evidence of an acidic vacuole content. Nitrate accumulation in Arabidopsis thaliana vacuoles is mediated by a 2-NO_3_^−^/H^+^ antiporter (AtCLCa) that is similar to widely distributed H^+^/Cl^−^ exchangers [66]. In the *Beggiatoa* genome we identified proteins (BgP0076 and BgP4800) related to H^+^/Cl^−^ exchangers (clcA), and chloride channels that display weak similarities to the AtCLCa antiporter.

### Dimethyl Sulfoxide and Sulfur Respiration

Flexibility in respiratory pathways is highly beneficial for organisms living under fluctuating environmental conditions such as can occur at sediment surfaces. As an alternative to nitrate and oxygen, *Beggiatoa* may also respire dimethyl sulfoxide (DMSO) to form the important anti-greenhouse gas dimethyl sulfide, as indicated by the presence of DMSO reductase genes *(dmsABC)* in the PS dataset. DMSO is frequently formed by eukaryotic plankton [68] and by photochemical oxidation of dimethyl sulfide [69]. Because DMSO is dissolved in sea water, *Beggiatoa* could access this alternative electron acceptor at the sediment surface. Additionally, the *Beggiatoa* genome encodes a thiosulfate reductase *(phsABC),* which is probably also involved in the reduction of elemental sulfur and tetrathionate [70]. Moreover, a thiosulfate reductase is also involved in disproportionation of thiosulfate [71], which is a significant intermediate in marine sulfur cycling [72]. The hypothesized inorganic sulfur reduction is in accordance with previous results in B. alba that have reported reduction of stored elemental sulfur under short-term anoxic conditions [73,74].

### Carbon Metabolism

Apart from one strain, all freshwater *Beggiatoa* require organic substrates for growth, in contrast to autotrophic marine *Beggiatoa* [16]. In our *Beggiatoa* the ability to fix carbon dioxide for autotrophic growth is encoded as a form I ribulose-bisphosphate carboxylase oxygenase (RubisCO), first reported for a non-vacuolated strain [75]. In addition, a phosphoribulokinase and a carbonic anhydrase gene are predicted. However, the non-vacuolated B. alba and B. leptomitiformis also grow heterotrophically using acetate and other organic compounds [76–78]. Earlier studies on marine, non-vacuolated strains have shown a broad spectrum of utilized organic compounds [79]. Similarly, our data suggest that the large vacuolated *Beggiatoa* and their relatives are also not obligate lithoautotrophs. Both genomes harbor acetate/cation symporters, acetate kinase, and putative acetyl-coenzyme A synthetase to channel acetate into the general metabolism. Accordingly, in the related *Thiomargarita,* sulfur oxidation was stimulated upon acetate amendment [80]. During growth on acetate, the glyoxylate cycle is probably employed for gluconeogenesis, as observed in other *Beggiatoa* [54,77]. However, the key enzymes malate synthase and isocitrate lyase were not identified in the incomplete genomic sequences. Several enzymes of the tricarbonic acid cycle were identified, such as isocitrate and succinate dehydrogenase. In contrast to the free-living gammaproteobacterial sulfur-oxidizer Thiomicrospira crunogena [81], *Beggiatoa* encodes a 2-oxoglurate dehydrogenase and a malate dehydrogenase, whereas fumarate dehydratase, PEP carboxylase, and succinyl-coenzyme A synthase are possibly encoded on the unsequenced part of the genome. In general, these findings are consistent with experimental results [54] and suggest the presence of a complete set of tricarbonic acid cycle enzymes.

Furthermore, the presence of three subunits of a glycolate oxidase *(glcDEF)* suggests a utilization of glycolate, which originates from photosynthetic organisms, e.g., co-occurring cyanobacteria. The presence of genes encoding poly-β-hydroxybutyric acid synthase, acetyl-coenzyme A acetyltransferase, and acetoacetyl-coenzyme A reductase is consistent with the observation of large, visible granules of poly-β-hydroxybutyric acid in *Beggiatoa* and relatives [16]. The synthesis of polyglucoses in *Beggiatoa* has not been previously reported, but both genome datasets point to the capability to synthesize glycogen preferentially under oxic conditions, as in *Thiomargarita* [14], as illustrated by genes encoding glycogen synthase and glycogen-debranching enzymes. *Beggiatoa* could also synthesize ATP via substrate-level phosphorylation from pyruvate via a probable fermentative lactate dehydrogenase *(ldh).* Fermentation of storage compounds and pyruvate enables *Beggiatoa* to persist during periods of oxygen, sulfur, and nitrate depletion, e.g., when the oxic–anoxic interface is located above the sediment surface.

### Phosphate Accumulation

**Figure 5:**
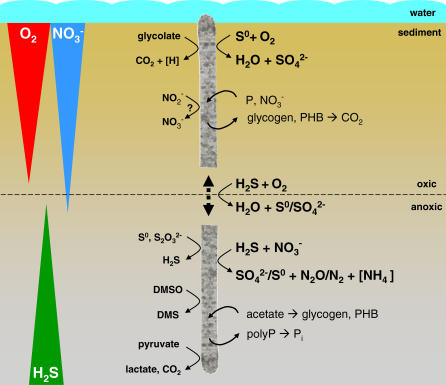
Scheme Summarizing Potential Energy-Yielding Pathways in *Beggiatoa* in Vertical Gradients of Oxygen, Nitrate, and Hydrogen Sulfide in Marine Surface Sediment Inspired by [11,14]. DMS, dimethyl sulfide; PHB, poly-β-hydroxybutyric acid.

Under nutritional imbalance many bacteria accumulate phosphate, which is intracellularly stored as polyP. *Thiomargarita* and *Thioploca* exhibit an efficient phosphate uptake and storage system and contain large polyP granules. Recently, these organisms were hypothesized to account for large phosphorite deposits at the sea floor [14]. In *Beggiatoa* the ability for polyP storage has not been unambiguously proven [16]. Here, we provide genetic evidence for polyP storage in *Beggiatoa*. Interestingly, both *Beggiatoa* datasets encode phytases. Phytate is an important inorganic phosphate storage compound in plants and adsorbs to particles in sediments and soils. The phytases likely enable *Beggiatoa* to access inorganic phosphate more efficiently. In addition, *Beggiatoa* takes up polyP and orthophosphate via selective porins O/P and high-affinity *phoBRU*-regulated ABC phosphate transporters. After uptake, a polyP kinase catalyzes the synthesis of polyP granules. In analogy to phosphorus removal from activated sludge, *Beggiatoa* and relatives may accumulate polyP at the sediment surface under aerobic conditions and degrade polyP under anaerobic conditions at the depth where they uptake acetate [14] (Figure 5).

### Secondary Metabolites

Unexpectedly, *Beggiatoa* appears to harbor the potential to synthesize secondary metabolites. We identified numerous genes of presumably cyanobacterial origin that encode non-ribosomal peptide synthetases and polyketide synthetases (PKS) (Table 2). Several functional domains are required for NRP and also for PK synthesis, respectively. Adenylation (AMP-A), acyltransferase (phosphopantetheine-binding), condensation, and thioesterase domains are present in the PS dataset (Table 2) and to a lesser extent in the SS dataset. The phylogenetic analysis of selected AMP A-type domains in *Beggiatoa* supports a mostly cyanobacterial origin of non-ribosomal peptide synthetases (Figure S4). The derived polypeptides show high similarities to proteins involved in synthesis of toxins and antibiotics rather than to fatty acid synthases. ORF BgP2814ff and downstream sequences (3,576 bp) display their highest similarities to anabaenopeptilide and nostopeptolide synthetases of Anabaena sp. and Nostoc sp., respectively, which are polyketide–non-ribosomal peptide hybrids of the microcystin family [82,83]. Other derived polypeptides of *Beggiatoa* (e.g., BgP5597 and BgP1194) exhibit significant similarities to modules of polyketide synthetases in Nostoc punctiforme. Since the presence of AMP-A domains in cyanobacteria is correlated with the synthesis of natural bio-active products [84], we hypothesize a similar capability to form secondary metabolites in *Beggiatoa*. These genetic findings have been corroborated by a HPLC-MS-based analysis of a methanol extract from a *Beggiatoa* mat from the sampling site that indicated a significant fraction of compounds of a molecular weight comparable to polyketides (S. Rachid, unpublished data).

**Table 2:**
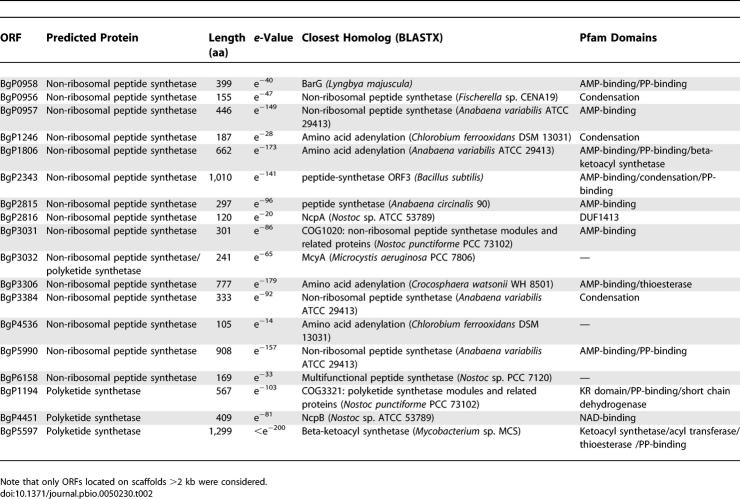
Predicted Protein Coding Sequences in the PS Genome Related to Secondary Metabolite Synthesis

### Exoproteins Related to Filamentous Cyanobacteria

We identified numerous ORFs that are homologous to large putative exoproteins, several of which contain a hemagglutination activity domain. Generally these glycoproteins are associated with cell adhesion and cell aggregation in biofilms of pathogenic bacteria [85]. Intriguingly, in *Beggiatoa* the derived proteins phylogenetically affiliate with the cyanobacterial genera *Nostoc, Anabaena,* and *Trichodesmium,* and also *Hahella chejuensis,* an exopolymer-producing gammaproteobacterium (Table S8; Figure S5). Similar to in cyanobacteria, several paralogs are encoded in the *Beggiatoa* genome, which may point to a functional relevance of the respective proteins. The striking similarity to filamentous, gliding cyanobacteria suggests a function of these proteins in gliding motility, and for sheath or filament formation. Indeed, glycoconjugates were recently detected in high amounts at the outer surface of *Beggiatoa* filaments using fluorescently labeled lectins (S. Hinck, unpublished data). Hence, the identified exocellular glycoproteins likely play a role in slime production, S-layer formation, or cell–cell adhesion.

### Conclusions

We have shown that the combination of optical mapping, WGA, and pyrosequencing offers great potential for genomic analysis of individual, uncultured bacteria. However, the incomplete sequence assemblies limited the accurate determination of the genome size and an in-depth analysis of the *Beggiatoa* genome. Generally, the contribution of non-target DNA cannot be completely ruled out in environmental WGA projects; thus, polyphasic approaches are indispensable to test for the purity of the assembled sequences. Keeping these methodological issues in mind, the genomic analysis of single *Beggiatoa* filaments has generated numerous novel hypotheses with regard to their ecophysiology and evolution that can now be experimentally tested. Breadth of storage capabilities and a highly flexible energy metabolism, together with gliding motility, optimally equip these large marine *Beggiatoa* to thrive under spatially and temporally fluctuating conditions at sediment surfaces. The striking similarity between numerous genes of *Beggiatoa* and cyanobacteria, along with their obvious shared phenotypic characteristics, points to pronounced horizontal gene transfer between these organisms, likely facilitated by the long-term coexistence of *Beggiatoa* and cyanobacteria in surface sediments and microbial mats [86].

## Materials and Methods

### Sampling and filament purification.

The Beggiatoa spp. filaments were obtained in Eckernförde Bay (Germany, Baltic Sea, 54° 47′ N/9° 83′ E). The surface of the *Beggiatoa*-covered sediment (∼4 m water depth) was sampled in August 2004 and December 2005 using polyacryl tubes. The sediment was kept in the dark at 4 °C until further processing. Two single *Beggiatoa* filaments with a diameter of 30 μm and length of ∼1 cm were transferred from the sediment surface to a Petri dish filled with artificial sea water medium containing agar. While gliding through the agar the *Beggiatoa* filaments were cleaned of particles and adhering bacteria.

### Bacterial cell lysis and DNA denaturation.

Purified filaments of *Beggiatoa* were individually lysed as follows. A filament was placed in 27 μl of TE (10 mM Tris-HCl [pH 7.2], 1 mM Na_2_ EDTA) and subjected to ten alternating cycles of freezing/thawing in a dry ice–ethanol bath for 1 min and thawing at room temperature to enhance cell lysis. The DNA was denatured by the addition of 3 μl of KOH (0.4 M) and EDTA (10 mM). The lysate was incubated at 65 °C in a water bath for 3 min, and neutralized with 3 μl of Tris-HCl (pH 4) according to [21].

### Amplification of Beggiatoa sp. DNA by WGA.

We employed MDA as a means of WGA to prepare sufficient DNA for genomic library construction and cloneless pyrosequencing. The REPLI-g kit (Qiagen; http://www.qiagen.com/) was used for MDA according to the manufacturer's instructions. Reactions contained 33 μl of the neutralized cell lysate and 25 μl of 4× MDA reaction mix, and were adjusted with water to a final volume of 100 μl. The reactions were incubated at 30 °C for 16 h and stopped by shifting to 65 °C for 3 min. The DNA concentration in the MDA product accumulated to a concentration of ∼1.4 mg/ml in all treatments.

### Clone library construction.

MDA-amplified genomic DNA of one filament was sheared using a Hydroshear instrument (Genomic Solutions; http://www.genomicsolutions.com/) with speed code set to two for 30 cycles to yield DNA fragments of a size mainly between 4 and 6 kb. The gel-purified MDA products were then cloned into the pCR4 TOPO vector (Invitrogen; http://www.invitrogen.com/). The ligation products were used to transform TOP10 Escherichia coli using the pCR4 Blunt-TOPO vector cloning kit (Invitrogen) according to the manufacturer's instructions. Transformants were plated on 22-cm^2^ Q-trays (Genetix; http://www.genetix.com/) containing 100 μg/ml kanamycin. Kanamycin-resistant colonies were then picked using a Q-bot (Genetix) and arrayed in 96-well microtiter plates.

### Sanger sequencing of the shotgun clone library.

Plasmids for sequencing were robotically extracted from overnight cultures using a RevPrep Orbit (Genomic Solutions) or a Biomek FX Liquid Handling Robot (Beckman Coulter; http://www.beckmancoulter.com/). DNA sequencing setups, cycle sequencing, and sequencing reaction clean-ups were all performed using a Parallab Nanoliter Pipetting Robot (Parallab; http://www.parallab.uib.no/). The labeling reactions were performed in a volume of 50 nl using ABI BigDye Cycle Sequencing kits (Applied Biosystems; http://www.appliedbiosystems.com/), the thermal cycling was performed in an integral air cycler, and the clean-ups were conducted in capillaries using magnetic beads. The sequencing reactions were then loaded onto an ABI 3730xl DNA Analyzer (Applied Biosystems) for capillary electrophoretic separation and calling of the sequencing products. Both ends of each clone were sequenced using vector-based primers to provide mate-pair information. Approximately 8,800 sequence reads were obtained, of which 4,700 were usable for assembly.

### Clone-free sequencing of MDA-amplified genomic DNA (Pyrosquencing).

The genomic DNA of a second, morphologically identical *Beggiatoa* filament was amplified using the MDA technique described above. The amplified DNA served as a template for sequencing using the clone-free pryosequencing technology developed by 454 Life Sciences (http://www.454.com/) [31]. Raw images from all regions of six-picotiter sequencing plates (one 60 × 60 and five 70 × 75) were processed with the three components (image processing, signal processing, and the Newbler de novo assembler) of the latest available version (1.0.51.03) of the 454 Life Sciences off-instrument data processing software to yield the PS assembly (Tables 1, S1, and S2). Additional sequencing was halted when the length of the all-contigs dataset did not increase with additional 454 Life Sciences sequencing runs, and we attribute the limited convergence of the length of the large-contig dataset (i.e., to the length of the all-contig dataset) to the unusually high percentage of repeat sequences in the MDA reaction product used for pyrosequencing. A subset (0.9 Mb; 3,448 fragments; length range 81–643 bases, each supported by at least ten reads) of the 22,858 small contigs produced by the 454 Life Sciences assembler was added to the large-contig dataset the assembler produced (6.7 Mb; 3,321 contigs, each >500 bases) to yield the 7.6-Mb PS assembly (Figure S8).

### Optical mapping.

For optical genome mapping, five *Beggiatoa* filaments 35 μm in diameter and >1 cm in length were purified as described above and immediately transferred into an agar drop containing cell suspension buffer (10 mM Tris-HCl [pH 7.2], 20 mM NaCl, 100 mM EDTA, 5 mg/ml freshly prepared lysozyme, 1% LMP agarose kept at 70 °C). After solidification at 4 °C the agar drop was incubated in cell lysis buffer (0.5 M EDTA, 1% laurosyl sarcosine, 2 mg/ml proteinase K [pH 9.5]) at 50 °C overnight. The determination of the chromosome size was performed as reported earlier [32].

### Gene prediction and annotation.

The DNA sequence data of the PS and SS approaches were each divided into two sub-databases. These sub-databases were used for the analysis of scaffolds of length <2 kb (PS1 and SS1) since ORF prediction on short fragments is not possible with standard ORF-finding tools, because of missing information. All scaffolds in these sub-databases were translated into all six reading frames and treated as artificial ORFs in the ongoing analysis to perform similarity searches. The second set of sub-databases consisted of all sequenced scaffolds longer than 2 kb for each approach (PS2 and SS2). All scaffolds in these databases were used for ORF prediction using the metagene prediction software MORFind (J. Waldmann and H. Teeling, unpublished data) developed at the Max Planck Institute for Marine Microbiology, Bremen. This system analyzes and combines the output of the three commonly used gene finders CRITICA, GLIMMER, and ZCURVE to enhance sensitivity and specificity. To resolve conflicts, an iterative post-processing algorithm is used, taking into account signal peptide and transmembrane predictions, ORF length, and the number of gene finders by which an ORF has been predicted.

Annotation was performed by a refined version of the GenDB v2.2 system [87], supplemented by the comparative analysis tool JCoast (http://www.megx.net/jcoast/) developed at the Max Planck Institute for Marine Microbiology, Bremen. For each predicted ORF the system retrieves observations from similarity searches against sequence databases NCBI-nr, Swiss-Prot, and KEGG GENES (release April 2006) and protein family databases Pfam (release 20.0) and InterPro (release 12.0, InterProScan v4.2), and from predictive signal peptide analysis (SignalP v3.0 [88]) and transmembrane helix analysis (TMHMM v2.0 [89]). tRNA genes were identified using tRNAScan-SE [90]. Predicted protein coding sequences were automatically annotated with the software MicHanThi [91] developed at the Max Planck Institute for Marine Microbiology, Bremen. The system simulates the reasoning in the human annotation process using fuzzy logic. The annotations of all ORFs described in this publication were manually refined.

### Phylogenetic best BLAST analysis.

To evaluate the phylogenetic consistency of the conserved ORFs in the databases PS2 and SSI2, all conserved ORFs were tested by BLAST analysis for the phylogenetic distribution of best hits against a local genome database (genomesDB; M. Richter, unpublished data). Only hits with an *e*-value below e^−05^ were considered significant. The local genome database (genomesDB) provides a computationally well-defined environment of 311 published whole genome sequences of bacterial and archaeal origin, with all ORFs of each genome carrying a unique ID. To allow genome comparisons between specific user-defined groups, all ORFs are assigned to the respective organism and metabolic group. In contrast to the general purpose database NCBI-nr, which contains every sequence ever submitted, the focus of genomesDB is the association of every protein to their phylogenetic affiliation in a refined environment.

### Cluster analysis.

For all sequences of the PS dataset the following intrinsic DNA signatures were calculated: (1) dinucleotide relative abundances [92], (2) Markov-model-based statistical evaluations of tri- and tetramer over- and underrepresentation [93], and (3) normalized chaos game representations for tri- and tetramers [94]. Values for (2) and (3) were computed by ocount and cgr, respectively, two self-written C-programs that are publicly available ( http://www.megx.net/tetra_new/html/download.html). The self-written Java program MetaClust [95] was used to automatically trigger the individual calculations and subsequently store them in a MySQL database. After that, MetaClust was also applied to build different combinations of subsets of the individual methods for all sequences exceeding 5 kb and trigger a hierarchical clustering of them using Cluster 3.0 [96]. For the clustering, complete linkage was used as the clustering algorithm, and the Euclidean distance was used as the distance measure. The corresponding result files were analyzed using Java TreeView (http://jtreeview.sourceforge.net/) and checked for outliers. This procedure was repeated for all sequences exceeding 4 kb, 3 kb, 2 kb, and 1kb and for all sequences of the dataset.

### Comparison of shared gene content by RBMs.

To compare the two datasets for shared genes we performed a “BLAST all against all” analysis between all predicted ORFs in the datasets PS2 and SS2. RBMs were counted only if the *e*-value was below the cut-off of e^−05^.

### Phylogenetic analysis.

All phylogenetic analyses were performed with the ARB/Silva software package ([97]; http://www.arb-silva.de/). The partial 16S rRNA gene sequences were inserted into a phylogenetic tree based on nearly complete sequences. The alignment was corrected manually. Phylogenetic trees were calculated by maximum parsimony, neighbor joining, and maximum likelihood analysis with different sets of filters. Topologies were evaluated to elaborate a consensus tree. Branching orders that were not supported by all methods are shown as multifurcations. Subsequently, partial sequences were inserted into the reconstructed tree by applying the parsimony criteria without allowing changes in the overall tree topology. Multiple alignments of protein sequences of nitrate reductase alpha subunits (NarG), AMP domains of non-ribosomal peptide synthetases, hemagglutination-domain-containing proteins (Hgg) were established with the ClustalW program package using the BLOSUM62 substitution matrix. For the phylogenetic analysis of NarG and Hgg maximum likelihood trees (Molphy, http://plone.jcu.edu.au/hpc/software-installation/molphy) were reconstructed using JTT amino acid substitution matrix for evolutionary distance. Distance matrix trees were calculated using the neighbor joining function of ARB with the Kimura correction for proteins. Different base frequency filters were applied. For phylogenetic reconstruction of AMP-A domains of non-ribosomal peptide synthetases, nearly full-length sequences were extracted. Maximum parsimony, neighbor joining, and PHYLIP distance matrix trees were calculated using different correction factors (see above). For calculations, 219 amino acid positions were considered, excluding major deletions and insertions. A set of 41 concatenated protein sequences were considered to determine the phylogenetic position of *Beggiatoa*. The following protein sequences were used for maximum parsimony, neighbor joining, and maximum likelihood trees: RNA polymerase (rpoC), recA, and ribosomal proteins L1–L5, L7/L12, L9–L11, L13–L24, L27–L29, L35, S2–S8, S11–S13, and S15–S20. A 30% positional conservation filter was used (5,857 positions) to exclude variable positions.

### Rhodamine 123 staining.

Single *Beggiatoa* filaments were incubated for 40 s in filter-sterilized seawater containing 200 μM of the lipophilic cation rhodamine 123 (Molecular Probes http://probes.invitrogen.com/). After loading, filaments were thoroughly washed with seawater, placed in an incubation chamber, and mounted on the stage of an Oz confocal microscope (Noran Instruments, http://www.thermo.com/). The light from an argon ion laser (488 nm; Omnichrome, http://www.mellesgriot.com/) was delivered to the cells via a 40× oil immersion plan apochromat objective (NA 1.4; Nikon Instruments, http://www.nikoninstruments.com/). Fluorescence emission light was directed through a 500-nm LP barrier filter (Chroma Technology, http://www.chroma.com/) and quantified using a photomultiplier tube at eight-bit resolution (Hamamatsu Photonics, http://www.hamamatsu.com/). Hardware and image acquisition were controlled by Intervision software (v1.5; Noran Instruments) running under IRIX 6.2 on an Indy workstation (SGI, http://www.sgi.com/). Images (512 × 480 pixels) were collected at 30 Hz with a pixel dwell time of 100 ns and averaged using a window of 32 ns in real time.

## Supporting Information

Figure S1 Whole Genome AflII Optical Map of *Beggiatoa* Sp. The consensus map was built from the shown underlying maps, obtained from individual DNA molecules, and represented here as multicolored arcs. The outermost color circle is the consensus map generated. Congruent restriction fragments shown in the consensus map are denoted by a common color. The total chromosome size is 7.4 Mb.
(1.1 MB PPT)

Figure S2 Phylogenetic Consensus Tree Based on 41 Concatenated Protein Sequences, Showing the Phylogenetic Positioning of *Beggiatoa* Sp. (PS Dataset) Scale bar represents 10% estimated sequence divergence.
(259 KB PDF)

Figure S3 Evolutionary Relationships of the *Beggiatoa* Sp. Hypothetical Protein BgP4037 (A) Alignment of the conserved hypothetical protein BgP4037 and related proteins (displayed are positions 59–174 according to the BgP4037 sequence). Sequences were aligned using ClustalW; similar residues are highlighted according to the BLOSUM62 matrix for evolutionary substitution.
(B) Gene neighborhood of BgP4037 illustrating the co-localization of nitrate reductase subunits *(narHJ)* and the putatively cyanobacteria-derived gene BgP4037.
(604 KB PPT)

Figure S4 Phylogenetic Consensus Tree of AMP-A Domains of Putative Non-Ribosomal Peptide Synthetases in the *Beggiatoa* PS2 Dataset For calculations based on distance matrix and maximum parsimony, 219 amino acid positions were considered. Scale bar corresponds to 10% estimated sequence divergence.
(287 KB PDF)

Figure S5 Phylogenetic Consensus Tree of Hemagglutinin-Domain-Containing Proteins of *Beggiatoa* (PS Dataset) and Related Sequences Only sequences containing a complete hemagglutination domain and with a cut-off *e*-value of e^−06^ were selected for calculations. The scale bar corresponds to 10% estimated sequence divergence.
(270 KB PDF)

Figure S6 Phylogenetic Reconstruction Based on the Nitrate Reductase Alpha Subunits (NarG) of *Beggiatoa* Sp. (PS and SS Genomes) The partial sequence of NarG (BgS0139) was subsequently inserted into a maximum likelihood tree. Note the close affiliation of NarG from both Beggiatoa sp. genome sequences. The scale bar corresponds to 10% estimated sequence divergence. The asterisk marks the *narG* gene of the PS dataset, which consists of two concatenated, non-overlapping contigs with >99% sequence coverage.
(277 KB PDF)

Figure S7 Nitrous Oxide Production in Sediments at the Hakon Mosby Mud Volcano Vacuolated, nitrate-storing *Beggiatoa* from sediment of the Hakon Mosby mud volcano [98] were exposed to acetylene, which blocks the last step of denitrification, resulting in formation of nitrous oxide instead of molecular nitrogen. During the treatment, nitrous oxide microprofiles were measured continuously as published previously [98]. *Beggiatoa* filaments were collected from the sediments and placed on an agar layer (2% in seawater) to avoid contact with sulfide. In *Beggiatoa* filaments with adhering sediment*,* nitrous oxide development was observed (in red). Sediment not covered with *Beggiatoa* did show very low concentrations of nitrous oxide formed (in blue). The filaments were then centrifuged at 25,000*g* for 10 min at 4 °C, leading to disruption of the large *Beggiatoa* cells, but not of the small prokaryotes. The fact that nitrous oxide production almost completely stopped (in black) suggests that *Beggiatoa* was mainly responsible for the observed nitrous oxide production.
(55 KB PPT)

Figure S8 Abundance of Contig Size Classes (PS Dataset) 7.6 Mb in total.
(41 KB PPT)

Table S1 Metadata for the PS *Beggiatoa* Genome Assembly and Other Center for Genomic Sciences Pyrosequencing Bacterial Genome Assemblies with Varying Degrees of Apparent Repetitive DNA Content Here the correlation between the percentage of 454 Life Sciences reads excluded from a 454 Life Sciences assembly due to repetitive DNA and the degree of closure of assemblies produced by the 454 Life Sciences assembler is shown. For example, the 454 Life Sciences assembler yielded a *Beggiatoa* PS assembly that has about 30 times the percentage of its 454 Life Sciences reads excluded from the assembly due to repetitive DNA compared to the 6.9-Mb 454 Life Sciences assembly (unpublished data) generated at the Center for Genomic Sciences for the Pseudomonas aeruginosa strain CGSPaOppa8 (11.4% versus 0.4%), and the *Beggiatoa* PS assembly also has about 23 times the number of 454 Life Sciences assembler-generated contigs than CGSPaOppa8 (26,179 versus 1,154).
(53 KB DOC)

Table S2 Metadata for the 454 Life Sciences Reads Obtained during the Pyrosequencing of MDA-Amplified *Beggiatoa* DNA During the pyrosequencing of MDA-amplified *Beggiatoa* DNA, 454 Life Sciences reads coming from repeat regions constituted an unusually high percentage (11.3%) of the total reads (Table S2). These repeat reads have the same average Phred-equivalent base-call quality value (25.4), and virtually identical GC content (39.0% versus 38.9%) and average read length (107 versus 105 bases) as the assembled reads (82.4% of total).
(28 KB DOC)

Table S3 Phylo-BLAST Analysis of ORFs in the *Beggiatoa* PS Dataset BLASTP-based phylogenetic affiliation of the best-hit organisms. Cut-off value e^−05^.
(38 KB DOC)

Table S4 Ribosomal Proteins Encoded in the PS Dataset (105 KB DOC)

Table S5 Single-Copy Genes I in *Beggiatoa* Sp. (PS Dataset) Here, 30 out of a maximal 35 marker genes [37] occurring in most prokaryotes were identified that usually occur only once per genome and are not subject to horizontal gene transfer. None of the identified genes were redundant. A single asterisk indicates consecutive ORFs of one gene on the same contig (suggesting a possible sequencing frameshift); a double asterisk indicates non-overlapping fragments of the same gene located on different contigs.
(90 KB DOC)

Table S6 Single-Copy Genes II in *Beggiatoa* Sp. (PS Dataset) This list displays 40 out of a maximal 55 marker genes that are present in many (but not most) organisms and do not occur in duplicates. None of the identified genes were redundant in the PS dataset.
(100 KB DOC)

Table S7 Amino-Acyl tRNA Synthetase Genes in *Beggiatoa* Sp. (PS Dataset) None of the identified genes were redundant. A single asterisk indicates consecutive ORFs of one gene on the same contig (suggesting a possible sequencing frameshift); a double asterisk indicates non-overlapping fragments of the same gene located on different contigs.
(77 KB DOC)

Table S8 Hemagglutination-Domain-Containing Genes in *Beggiatoa* Sp. (PS Dataset) Amino acid similarities were calculated using the BLOSUM62 matrix.
(41 KB DOC)

### Accession Numbers

This whole genome shotgun project has been deposited at DNA Data Bank of Japan (http://www.ddbj.nig.ac.jp/), the EMBL Nucleotide Sequence Database (http://www.ebi.ac.uk/embl/), and GenBank (http://www.ncbi.nlm.nih.gov/Genbank/) under the project accessions ABBY00000000 (Beggiatoa sp. SS dataset) and ABBZ00000000 (Beggiatoa sp. PS dataset), respectively. The version described in this paper is the first version.

## Abbreviations

DMSO: dimethyl sulfoxide
MDA: multiple displacement amplification
ORF: open reading frame
polyP: polyphosphate
PS: pyrosequenced
RBM: reciprocal best match
SS: Sanger sequenced
WGA: whole genome amplification
