# Supplementary material for: Insights into the Genome of Large Sulfur Bacteria Revealed by Analysis of Single Filaments
Source: PLoS Biol. 2007 Aug 28;5(9):e230. doi: 10.1371/journal.pbio.0050230 (PMC1951784; doi:10.1371/journal.pbio.0050230)
Supplement: Supplementary file 1 [file pbio.0050230.sg002.pdf]

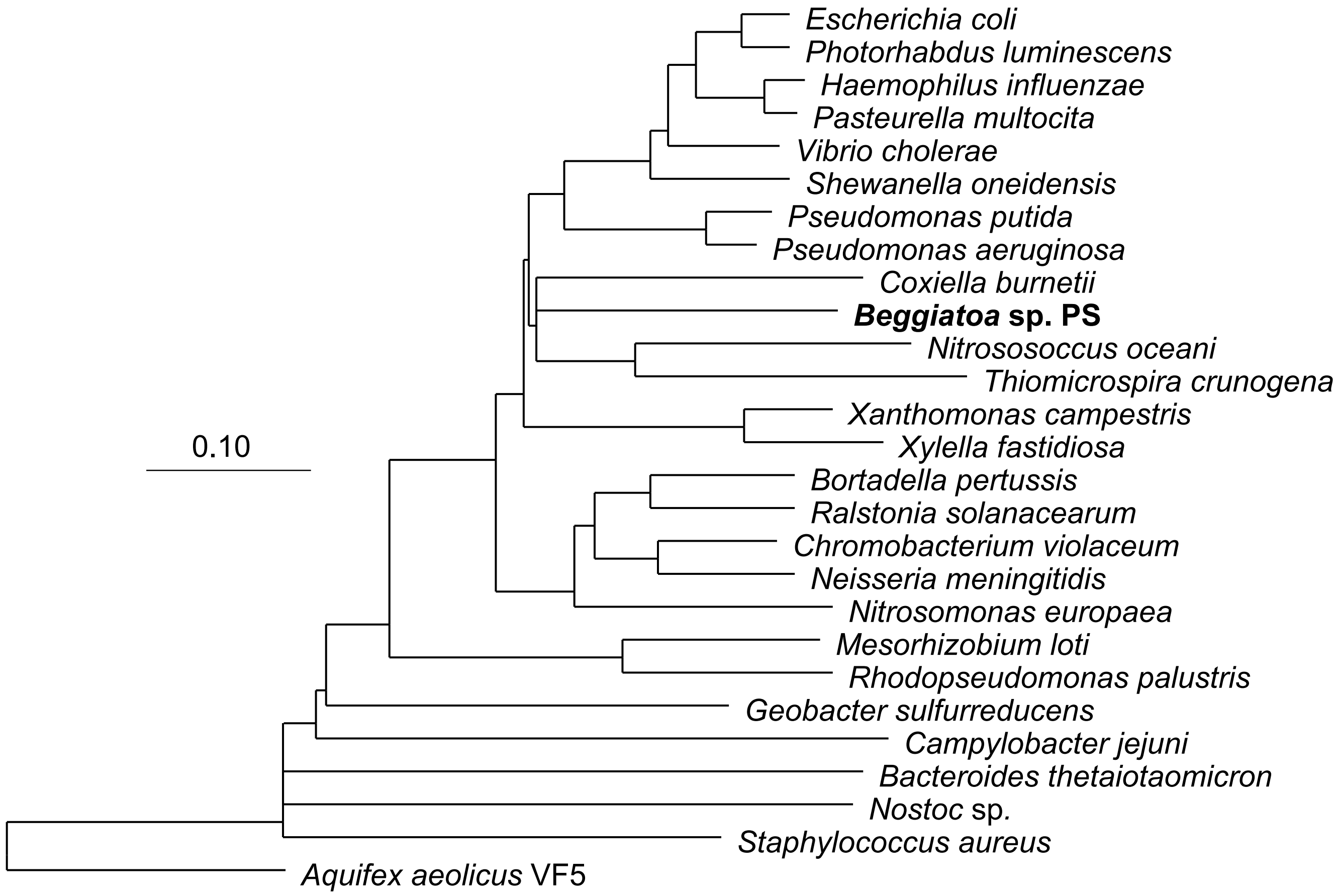

Escherichia coli
Photorhabdus luminescens
Haemophilus influenzae
Pasteurella multocita
Vibrio cholerae
Shewanella oneidensis
Pseudomonas putida
Pseudomonas aeruginosa
Coxiella burnetii
Beggiatoa sp. PS
Nitrososoccus oceani
Thiomicrospira crunogena
Xanthomonas campestris
Xylella fastidiosa
Bortadella pertussis
Ralstonia solanacearum
Chromobacterium violaceum
Neisseria meningitidis
Nitrosomonas europaea
Mesorhizobium loti
Rhodopseudomonas palustris
Geobacter sulfurreducens
Campylobacter jejuni
Bacteroides thetaiotaomicron
Nostoc sp.
Staphylococcus aureus
Aquifex aeolicus VF5
0.10
