# Supplementary material for: Insights into the Genome of Large Sulfur Bacteria Revealed by Analysis of Single Filaments
Source: PLoS Biol. 2007 Aug 28;5(9):e230. doi: 10.1371/journal.pbio.0050230 (PMC1951784; doi:10.1371/journal.pbio.0050230)
Supplement: Supplementary file 2 [file pbio.0050230.sg004.pdf]

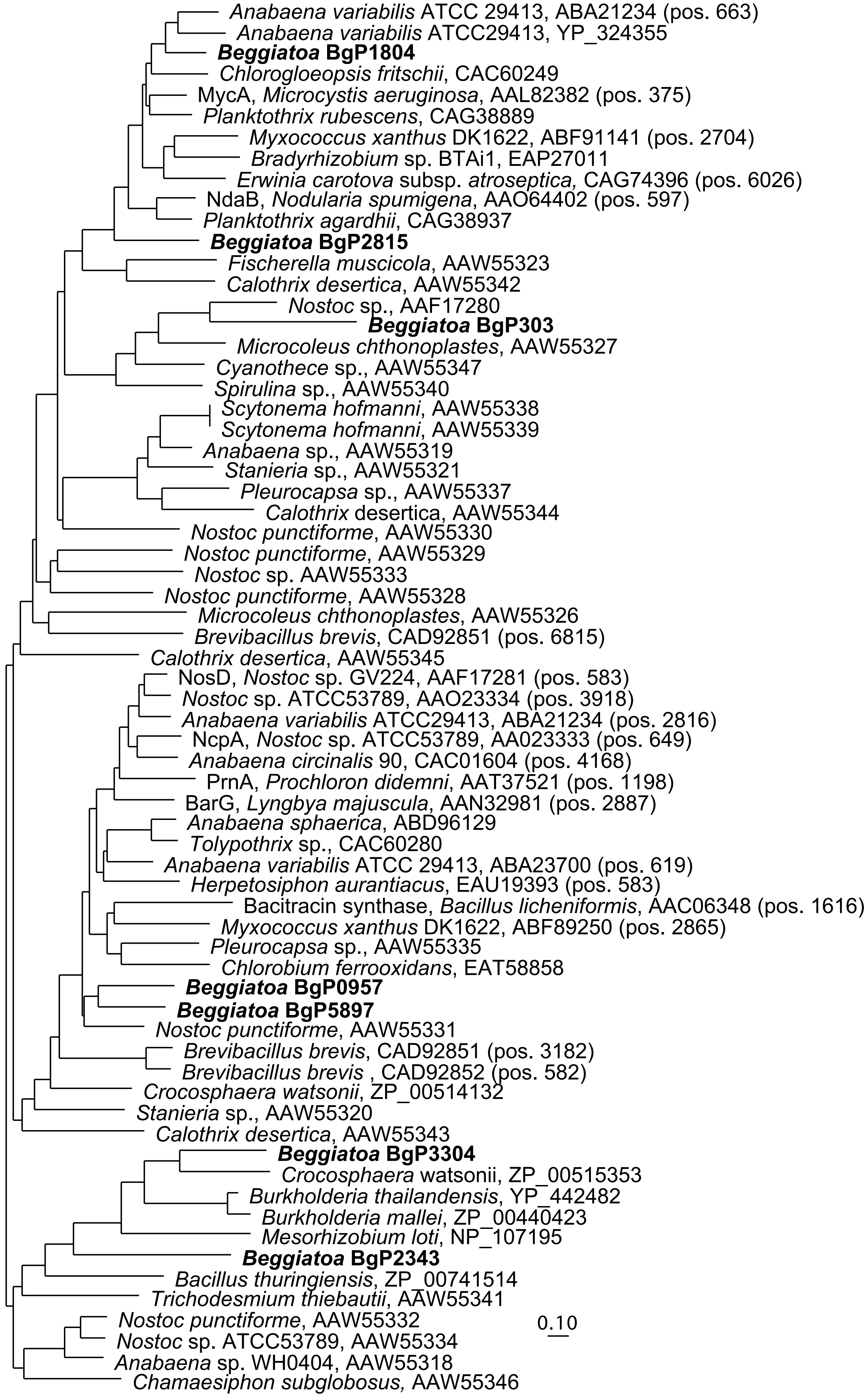
Anabaena variabilis ATCC 29413, ABA21234 (pos. 663)
Anabaena variabilis ATCC29413, YP_324355
Beggiatoa BgP1804
Chlorogloeopsis fritschii, CAC60249
MycA, Microcystis aeruginosa, AAL82382 (pos. 375)
Planktothrix rubescens, CAG38889
Myxococcus xanthus DK1622, ABF91141 (pos. 2704)
Bradyrhizobium sp. BTAi1, EAP27011
Erwinia carotova subsp. atroseptica, CAG74396 (pos. 6026)
NdaB, Nodularia spumigena, AAO64402 (pos. 597)
Planktothrix agardhii, CAG38937
Beggiatoa BgP2815
Fischerella muscicola, AAW55323
Calothrix desertica, AAW55342
Nostoc sp., AAF17280
Beggiatoa BgP303
Microcoleus chthonoplastes, AAW55327
Cyanothece sp., AAW55347
Spirulina sp., AAW55340
Scytonema hofmanni, AAW55338
Scytonema hofmanni, AAW55339
Anabaena sp., AAW55319
Stanieria sp., AAW55321
Pleurocapsa sp., AAW55337
Calothrix desertica, AAW55344
Nostoc punctiforme, AAW55330
Nostoc punctiforme, AAW55329
Nostoc sp. AAW55333
Nostoc punctiforme, AAW55328
Microcoleus chthonoplastes, AAW55326
Brevibacillus brevis, CAD92851 (pos. 6815)
Calothrix desertica, AAW55345
NosD, Nostoc sp. GV224, AAF17281 (pos. 583)
Nostoc sp. ATCC53789, AAO23334 (pos. 3918)
Anabaena variabilis ATCC29413, ABA21234 (pos. 2816)
NcpA, Nostoc sp. ATCC53789, AA023333 (pos. 649)
Anabaena circinalis 90, CAC01604 (pos. 4168)
PrnA, Prochloron didemni, AAT37521 (pos. 1198)
BarG, Lyngbya majuscula, AAN32981 (pos. 2887)
Anabaena sphaerica, ABD96129
Tolypothrix sp., CAC60280
Anabaena variabilis ATCC 29413, ABA23700 (pos. 619)
Herpetosiphon aurantiacus, EAU19393 (pos. 583)
Bacitracin synthase, Bacillus licheniformis, AAC06348 (pos. 1616)
Myxococcus xanthus DK1622, ABF89250 (pos. 2865)
Pleurocapsa sp., AAW55335
Chlorobium ferrooxidans, EAT58858
Beggiatoa BgP0957
Beggiatoa BgP5897
Nostoc punctiforme, AAW55331
Brevibacillus brevis, CAD92851 (pos. 3182)
Brevibacillus brevis , CAD92852 (pos. 582)
Crocosphaera watsonii, ZP_00514132
Stanieria sp., AAW55320
Calothrix desertica, AAW55343
Beggiatoa BgP3304
Crocosphaera watsonii, ZP_00515353
Burkholderia thailandensis, YP_442482
Burkholderia mallei, ZP_00440423
Mesorhizobium loti, NP_107195
Beggiatoa BgP2343
Bacillus thuringiensis, ZP_00741514
Trichodesmium thiebautii, AAW55341
Nostoc punctiforme, AAW55332
Nostoc sp. ATCC53789, AAW55334
Anabaena sp. WH0404, AAW55318
Chamaesiphon subglobosus, AAW55346
0.10
