# Supplementary material for: Insights into the Genome of Large Sulfur Bacteria Revealed by Analysis of Single Filaments
Source: PLoS Biol. 2007 Aug 28;5(9):e230. doi: 10.1371/journal.pbio.0050230 (PMC1951784; doi:10.1371/journal.pbio.0050230)
Supplement: Supplementary file 3 [file pbio.0050230.sg005.pdf]

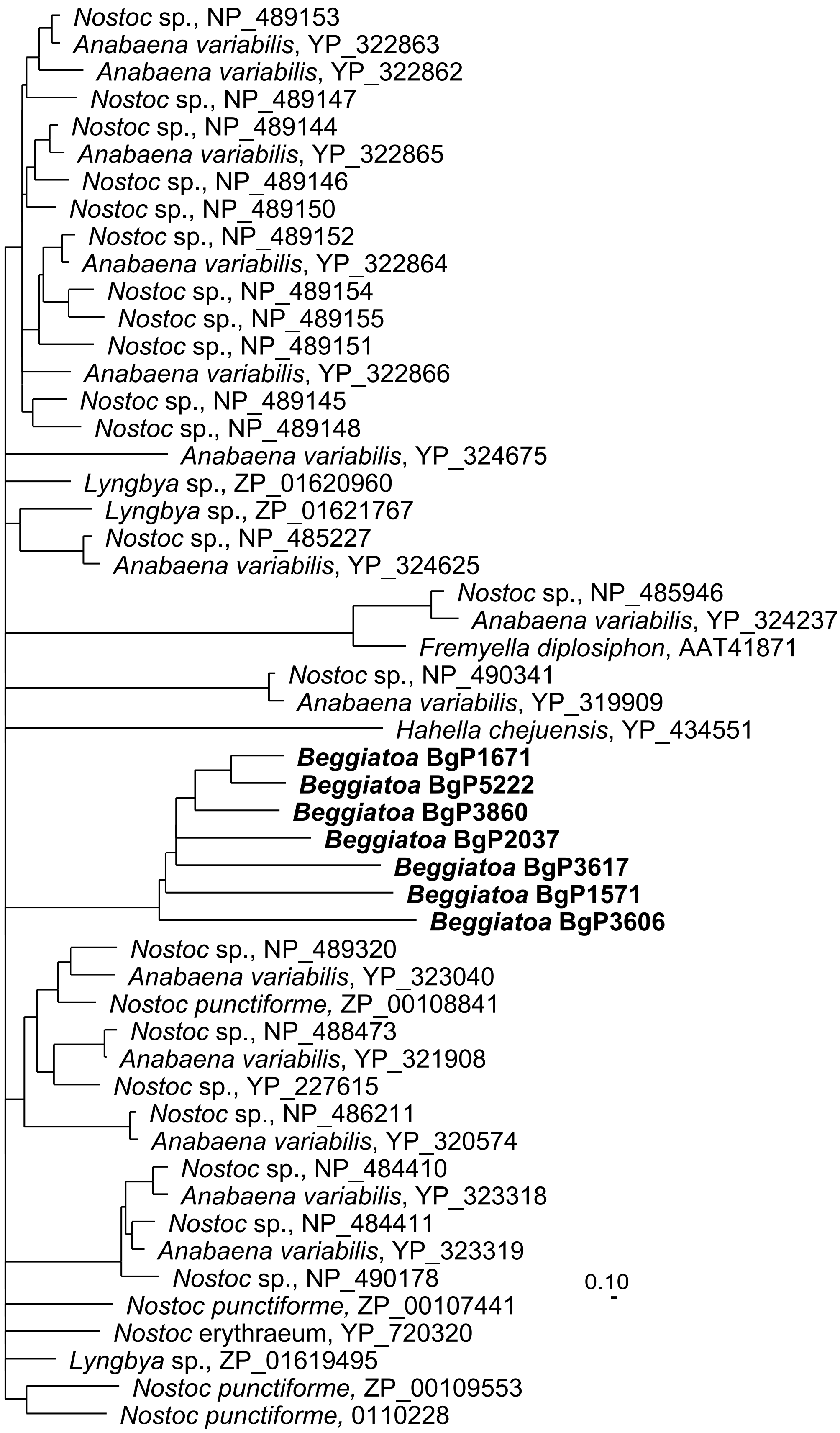
Nostoc sp., NP_489153
Anabaena variabilis, YP_322863
Anabaena variabilis, YP_322862
Nostoc sp., NP_489147
Nostoc sp., NP_489144
Anabaena variabilis, YP_322865
Nostoc sp., NP_489146
Nostoc sp., NP_489150
Nostoc sp., NP_489152
Anabaena variabilis, YP_322864
Nostoc sp., NP_489154
Nostoc sp., NP_489155
Nostoc sp., NP_489151
Anabaena variabilis, YP_322866
Nostoc sp., NP_489145
Nostoc sp., NP_489148
Anabaena variabilis, YP_324675
Lyngbya sp., ZP_01620960
Lyngbya sp., ZP_01621767
Nostoc sp., NP_485227
Anabaena variabilis, YP_324625
Nostoc sp., NP_485946
Anabaena variabilis, YP_324237
Fremyella diplosiphon, AAT41871
Nostoc sp., NP_490341
Anabaena variabilis, YP_319909
Hahella chejuensis, YP_434551
Beggiatoa BgP1671
Beggiatoa BgP5222
Beggiatoa BgP3860
Beggiatoa BgP2037
Beggiatoa BgP3617
Beggiatoa BgP1571
Beggiatoa BgP3606
Nostoc sp., NP_489320
Anabaena variabilis, YP_323040
Nostoc punctiforme, ZP_00108841
Nostoc sp., NP_488473
Anabaena variabilis, YP_321908
Nostoc sp., YP_227615
Nostoc sp., NP_486211
Anabaena variabilis, YP_320574
Nostoc sp., NP_484410
Anabaena variabilis, YP_323318
Nostoc sp., NP_484411
Anabaena variabilis, YP_323319
Nostoc sp., NP_490178
0.10
Nostoc punctiforme, ZP_00107441
Nostoc erythraeum, YP_720320
Lyngbya sp., ZP_01619495
Nostoc punctiforme, ZP_00109553
Nostoc punctiforme, 0110228
