# Supplementary material for: Insights into the Genome of Large Sulfur Bacteria Revealed by Analysis of Single Filaments
Source: PLoS Biol. 2007 Aug 28;5(9):e230. doi: 10.1371/journal.pbio.0050230 (PMC1951784; doi:10.1371/journal.pbio.0050230)
Supplement: Supplementary file 4 [file pbio.0050230.sg006.pdf]

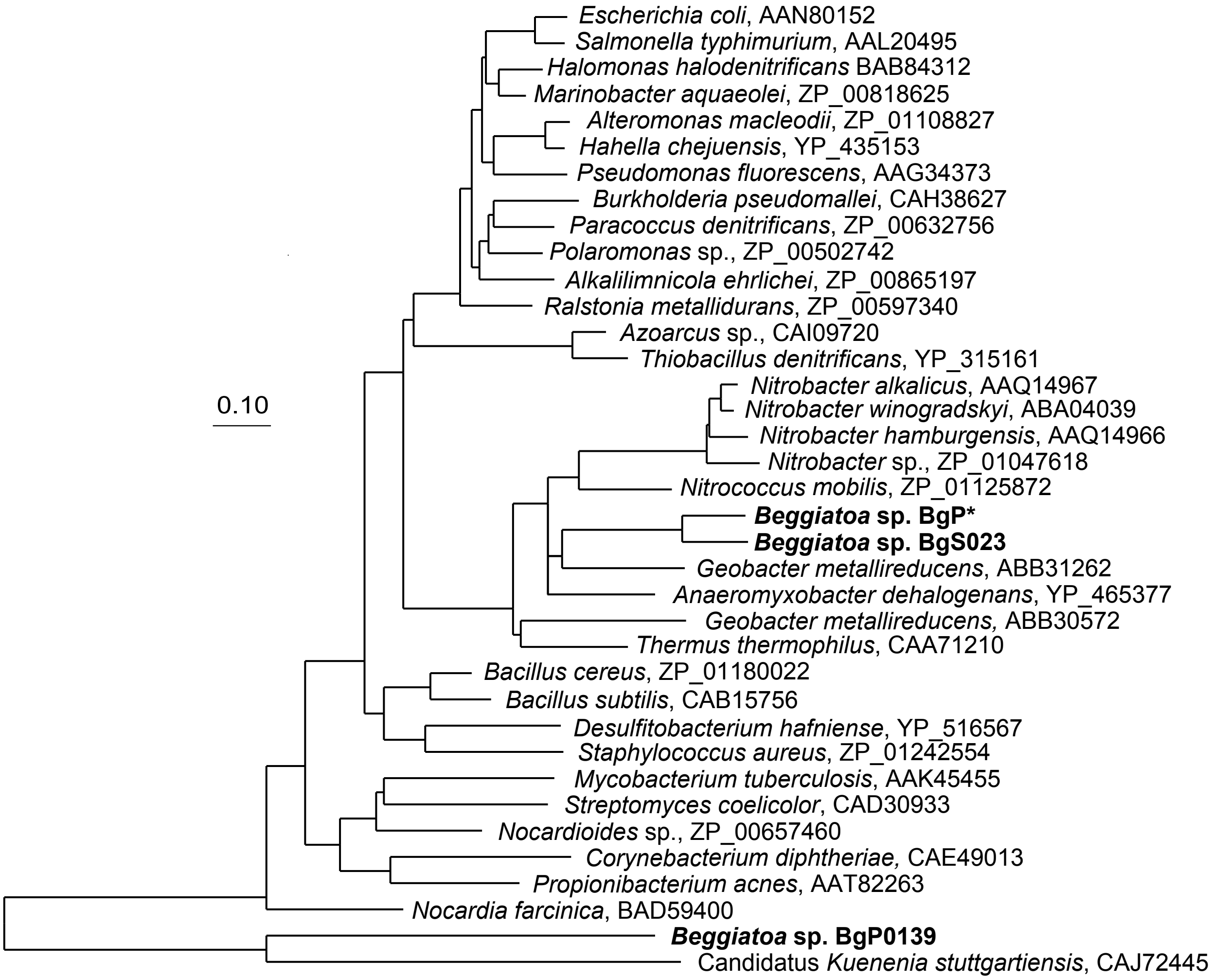
Escherichia coli, AAN80152
Salmonella typhimurium, AAL20495
Halomonas halodenitrificans BAB84312
Marinobacter aquaeolei, ZP_00818625
Alteromonas macleodii, ZP_01108827
Hahella chejuensis, YP_435153
Pseudomonas fluorescens, AAG34373
Burkholderia pseudomallei, CAH38627
Paracoccus denitrificans, ZP_00632756
Polaromonas sp., ZP_00502742
Alkalilimnicola ehrlichei, ZP_00865197
Ralstonia metallidurans, ZP_00597340
Azoarcus sp., CAI09720
Thiobacillus denitrificans, YP_315161
0.10
Nitrobacter alkalicus, AAQ14967
Nitrobacter winogradskyi, ABA04039
Nitrobacter hamburgensis, AAQ14966
Nitrobacter sp., ZP_01047618
Nitrococcus mobilis, ZP_01125872
Beggiatoa sp. BgP*
Beggiatoa sp. BgS023
Geobacter metallireducens, ABB31262
Anaeromyxobacter dehalogenans, YP_465377
Geobacter metallireducens, ABB30572
Thermus thermophilus, CAA71210
Bacillus cereus, ZP_01180022
Bacillus subtilis, CAB15756
Desulfitobacterium hafniense, YP_516567
Staphylococcus aureus, ZP_01242554
Mycobacterium tuberculosis, AAK45455
Streptomyces coelicolor, CAD30933
Nocardioides sp., ZP_00657460
Corynebacterium diphtheriae, CAE49013
Propionibacterium acnes, AAT82263
Nocardia farcinica, BAD59400
Beggiatoa sp. BgP0139
Candidatus Kuenenia stuttgartiensis, CAJ72445
