# Supplementary material for: Insights into the Genome of Large Sulfur Bacteria Revealed by Analysis of Single Filaments
Source: PLoS Biol. 2007 Aug 28;5(9):e230. doi: 10.1371/journal.pbio.0050230 (PMC1951784; doi:10.1371/journal.pbio.0050230)
Supplement: Supplementary file 5 [file pbio.0050230.st001.doc]

Table S1: Metadata^[a]^ for the pyrosequencing-based (PS) *Beggiatoa* genome

assembly and other CGS pyrosequencing bacterial genome assemblies with varying

degrees of apparent repetitive DNA content.

| **Line**  **[Note]** | **Property** | **PS data** | **Other CGS pyrosequencing bacterial genome assemblies** | | | | |
| --- | --- | --- | --- | --- | --- | --- | --- |
|  |  |  | PA | HI-max | HI-min | SP-max | SP-min |
| 01 | **Assembly** |  |  |  |  |  |  |
| 02 | GC% | 39 | 66 | 38 | 38 | 40 | 40 |
| 03 | length, total (Mb) | 7.6 | 6.9 | 1.9 | 1.8 | 2.0 | 2.0 |
| 04^[b]^ | coverage, average (fold) | 17 | 17 | 18 | 21 | 15 | 22 |
| 05 | contigs, count [lines 10 + 11] | 6,769 | 441 | 269 | 39 | 308 | 149 |
| 06 | **454Contigs** |  |  |  |  |  |  |
| 07 | length, total, all (Mb) | 9.8 | 7.0 | 2.1 | 1.8 | 2.1 | 2.1 |
| 08 | count, all | 26,179 | 1154 | 2040 | 142 | 509 | 341 |
| 09 | count, small | 22,858 | 713 | 1771 | 103 | 201 | 192 |
| 10 | count, large | 3,321 | 441 | 269 | 39 | 308 | 149 |
| 11^[c]^ | count, small, used in assembly | 3,448 | 0 | 0 | 0 | 0 | 0 |
| 12 | **454Reads, length** |  |  |  |  |  |  |
| 13 | total (Mb) | 219 | 120 | 39 | 38 | 33 | 46 |
| 14 | average (bases) | 106 | 99 | 108 | 107 | 105 | 105 |
| 15 | **454Reads, count, % of all reads** |  |  |  |  |  |  |
| 16 | assembled | 82.4 | 98.4 | 94.8 | 98.4 | 96.8 | 96.9 |
| 17 | repeat | 11.3 | 0.4 | 2.3 | 0.4 | 2.3 | 2.0 |
| 18 | singleton | 5.1 | 0.6 | 2.0 | 0.5 | 0.3 | 0.4 |
| 19 | outlier | 1.2 | 0.7 | 1.0 | 0.6 | 0.5 | 0.6 |
|  |  |  |  |  |  |  |  |

^[a]^ The Center for Genomic Sciences (CGS) has sequenced dozens of bacterial genomes (see www.ncbi.nlm.nih.gov/genomes/lproks.cgi) and produced draft assemblies using the “Newbler” assembler (which generated the metadata shown) and the technology developed by 454 Life Sciences, Inc. (“454”). Newbler output includes a set of “all contigs” and a subset thereof called “large contigs”, the latter typically >500 bases long. The set of 454 “all contigs” is assembled from two types of “reads” (actually, flowgrams): those termed “assembled” (line 16) and “partial” (partially assembled, typically insignificant in number). There are also three other types of 454 “reads”, all of which are excluded from an assembly: “singleton”, “repeat”, and “outlier”. The other genome assemblies shown are for *Pseudomonas aeruginosa* strain CGSPaOppa8 (PA); *Haemophilus influenzae* strains CGSHi22124 (HI-max) and CGSHiCC (Hi-min); and *Streptococcus pneumoniae* strains CGSSp14BS69 (SP-max) and CGSSp23BS72 (Sp-min). Assemblies of the genomes of these strains were either the closest in size to the PS assembly (PA), or represented assemblies that yielded the largest number (XX-max) or the smallest number (XX-min) of 454 large contigs for all of the genome assemblies for the indicated species, i.e., of the 12 or 8 genome assemblies for HI or SP (respectively) that have been sequenced to date at the CGS.

^[b]^ (number of “assembled” 454 reads) *(line 14)/(line 07).

^[c]^ A subset of small contigs, each of length > 70 bases and each supported by at least 10 reads.
