# Supplementary material for: Insights into the Genome of Large Sulfur Bacteria Revealed by Analysis of Single Filaments
Source: PLoS Biol. 2007 Aug 28;5(9):e230. doi: 10.1371/journal.pbio.0050230 (PMC1951784; doi:10.1371/journal.pbio.0050230)
Supplement: Supplementary file 6 [file pbio.0050230.st002.doc]

Table S2: Metadata^[a]^ for the 454 reads obtained during the pyrosequencing

of MDA-amplified *Beggiatoa* DNA

| **Type^[b]^** | **count (%)** | **count** | **length, avg.** | **length, total** | **base quality, avg.** | **GC%** |
| --- | --- | --- | --- | --- | --- | --- |
| assembled | 82.4 | 1,592,395 | 105 | 168,125,158 | 25.4 | 38.9 |
| repeat | 11.3 | 218,736 | 107 | 23,616,214 | 25.4 | 39.0 |
| singleton | 5.1 | 98,429 | 100 | 9,863,290 | 25.1 | 42.5 |
| outlier | 1.2 | 22,683 | 105 | 2,389,793 | 25.4 | 38.9 |
| partial | 0.0 | 67 | 96 | 6437 | 25.0 | 40.2 |

^[a]^ All of the 454 sequencing read and Phred-equivalent base-call quality data obtained during the pyrosequencing of *Beggiatoa* was imported into a relational database which was subsequently queried to obtain the metadata shown. For a given called base in a sequencing read, the Phred quality value = -10 * log_10_ (called base error probability). Thus, an average Phred-equivalent base-call quality value of 25.4 corresponds to an average expected probability of one error per 347 called bases in the 454 sequencing read data. Note the important observation in the 454 Life Sciences GS20 Data Processing Manual: “…there is no industry standard file format for representing the likelihood of an uncalled base: Quality scores should strictly be treated as the probability that individual called bases are correct in the sequence, with the understanding that they provide no information on the probability that a homopolymer stretch (i.e., at the end of which a called base may reside) might be longer than called.”

^[b]^ Definitions taken verbatim from the 454 Life Sciences GS20 Data Processing Manual:

“Assembled”-the read is fully incorporated into the assembly.

“Partially Assembled”-only part of the read was included in the assembly; the rest was deemed to have diverged sufficiently to not be included.

“Singleton”-the read did not overlap with any other reads in the input.

“Repeat”-the read was identified by the assembler as likely coming from a repeat region, and so was excluded from the final contigs.

“Outlier”-the read was identified by the assembler as problematic, and was excluded from the final contigs (one explanation of these outliers are chimeric sequences, but sequences may be identified as outliers simply as an assembler artifact).
