# Supplementary material for: Insights into the Genome of Large Sulfur Bacteria Revealed by Analysis of Single Filaments
Source: PLoS Biol. 2007 Aug 28;5(9):e230. doi: 10.1371/journal.pbio.0050230 (PMC1951784; doi:10.1371/journal.pbio.0050230)
Supplement: Supplementary file 7 [file pbio.0050230.st003.doc]

Table S3: Phylogenetic affiliation of ORFs based on BlastP analysis (PS2 dataset)

| **Phylum** | **No. of genes*** |
| --- | --- |
| *Proteobacteria* | 2654 |
| *Cyanobacteria* | 644 |
| *Firmicutes* | 162 |
| *Euryarchaeota* | 133 |
| *Chlorobi* | 104 |
| *Bacteroidetes* | 98 |
| *Planctomycetes* | 92 |
| *Actinobacteria* | 69 |
| *Spirochaetes* | 46 |
| *Fusobacteria* | 14 |
|  |  |
| *Nitrosococcus oceani* | 426 |
| *Nostoc* sp. | 213 |
| *Methylococcus capsulatus* | 204 |
| *Anabaena variabilis* | 194 |
| *Hahella chejuensis* | 172 |
| *Thiobacillus denitrificans* | 120 |
| *Chlorobium chlorochromatii* | 78 |

* of 4430 ORFs in total (PS2 dataset)
