# Supplementary material for: Insights into the Genome of Large Sulfur Bacteria Revealed by Analysis of Single Filaments
Source: PLoS Biol. 2007 Aug 28;5(9):e230. doi: 10.1371/journal.pbio.0050230 (PMC1951784; doi:10.1371/journal.pbio.0050230)
Supplement: Supplementary file 8 [file pbio.0050230.st004.doc]

| **Orf** | **predicted protein** | **Length (aa)** | **% residues aligned** | **e-value** | **Closest homolog (BLASTx)** | **Pfam domains** |  |
| --- | --- | --- | --- | --- | --- | --- | --- |
| BgP1035 | Ribosomal protein L14 | 122 | 100 | e-44 | ribosomal protein L14 [Nitrococcus mobilis Nb-231] | Ribosomal protein L14p/L23e |  |
| BgP2892 | Ribosomal protein S12/S23 | 127 | 97 | e-58 | Ribosomal protein S12 [Nitrosococcus oceani ATCC 19707] | Ribosomal protein S12 |  |
| BgP3490 | Ribosomal protein L27 | 116 | 60 | e-25 | Ribosomal protein L27 [Marinobacter aquaeolei VT8] | Ribosomal L27 protein |  |
| BgP5474 | ribosomal protein L4/L1e | 218 | 92 | e-51 | 50S ribosomal subunit protein L4 [Erwinia carotovora] | Ribosomal protein L4/L1 family |  |
| BgP5322 | Ribosomal protein S13 | 118 | 99 | e-45 | 30S ribosomal protein S13 [Francisella tularensis] | Ribosomal protein S13/S18 |  |
| BgP1057 | Ribosomal protein S6 | 125 | 91 | e-41 | 30S ribosomal protein S6 [Vibrio angustum S14] | Ribosomal protein S6 |  |
| BgP5473 | 50S ribosomal protein L23 | 100 | 98 | e-18 | Ribosomal protein L23 [Idiomarina loihiensis L2TR] | Ribosomal protein L23 |  |
| BgP2378 | Ribosomal protein S18 | 75 | 99 | e-30 | 30S ribosomal protein S18 [Nitrococcus mobilis Nb-231] | Ribosomal protein S18 |  |
| BgP4924 | 50S ribosomal protein L7/L12 | 124 | 100 | e-35 | Ribosomal protein L7/L12 [Nitrosococcus oceani ATCC 19707] | Ribosomal protein L7/L12 C-terminal domain | |
| BgP3017 | Ribosomal protein S16 | 200 | 44 | e-27 | ribosomal protein S16 [Alkalilimnicola ehrlichei MLHE-1] | Ribosomal protein S16 |  |
| BgP5320 | 30S ribosomal protein S4 | 206 | 100 | e-73 | 30S ribosomal protein S4 [Methylococcus capsulatus str. Bath] | Ribosomal protein S4/S9 N-terminal domain |  |
| BgP1031 | 30S ribosomal protein S3 | 211 | 99 | e-91 | ribosomal protein S3 [Alkalilimnicola ehrlichei MLHE-1] | Ribosomal protein S3, C-terminal domain |  |
| BgP2891 | Ribosomal protein S7 | 156 | 100 | e-54 | Ribosomal protein S7 [Nitrosococcus oceani ATCC 19707] | Ribosomal protein S7p/S5e |  |
| BgP1029 | Ribosomal protein S19 | 92 | 100 | e-36 | 30S ribosomal protein S19 [Vibrio splendidus 12B01] | Ribosomal protein S19 |  |
| BgP3491 | Ribosomal protein L21 | 184 | 54 | e-17 | 50S ribosomal protein L21 [Alcanivorax borkumensis SK2] | Ribosomal prokaryotic L21 protein |  |
| BgP4150 | Ribosomal protein L31 | 72 | 89 | e-23 | Ribosomal protein L31 [Nitrosococcus oceani ATCC 19707] | Ribosomal protein L31 |  |
| BgP1030 | Ribosomal protein L22 | 109 | 100 | e-38 | Ribosomal protein L22 [Shewanella sp. PV-4] | Ribosomal protein L22p/L17e |  |
| BgP5321 | Ribosomal protein S11 | 129 | 100 | e-55 | 30S ribosomal subunit protein S11 [Escherichia coli K12] | Ribosomal protein S11 |  |
| BgP2960 | Ribosomal protein S21 | 72 | 60 | e-14 | 30S ribosomal subunit protein S21 [Escherichia coli K12] | Ribosomal protein S21 |  |
| BgP4925 | Ribosomal protein L10 | 179 | 97 | e-53 | ribosomal protein L10 [Methylococcus capsulatus str. Bath] | Ribosomal protein L10 |  |
| BgP4936 | Ribosomal protein S2 | 260 | 85 | e-91 | 30S ribosomal protein S2 [Thiomicrospira crunogena XCL-2] | Ribosomal protein S2 |  |
| BgP2470 | Ribosomal protein S30EA | 108 | 95 | e-36 | Ribosomal protein S30EA [Nitrosococcus oceani] | Sigma 54 modulation protein / S30EA |  |
| BgP0484 | Ribosomal protein L33 | 51 | 98 | e-12 | ribosomal protein L33 [Oceanospirillum sp. MED92] | Ribosomal protein L33 |  |
| BgP2659 | Ribosomal protein S5 | 170 | 99 | e-53 | 30S ribosomal protein S5 [Congregibacter litoralis KT71] | Ribosomal protein S5, N-terminal domain |  |
| BgP6073 | Ribosomal protein L28 | 78 | 100 | e-30 | 50S ribosomal subunit protein L28 [Saccharophagus degradans 2-40] | Ribosomal L28 family |  |
| BgP1821 | Ribosomal protein L25 | 212 | 90 | e-47 | Ribosomal protein L25 [Idiomarina baltica OS145] | Ribosomal L25p family |  |
| BgP1028 | Ribosomal protein L2 | 224 | 100 | e-93 | Single-strand binding protein [Saccharophagus degradans] | Ribosomal Proteins L2, C-terminal domain |  |
| BgP3103 | Ribosomal protein L19 | 122 | 96 | e-42 | ribosomal protein L19 [Vibrio cholerae] | Ribosomal protein L19 |  |
| BgP3869 | Ribosomal protein L1 | 201 | 100 | e-72 | 50S ribosomal protein L1 [Methylococcus capsulatus str. Bath] | Ribosomal protein L1p/L10e family |  |
| BgP1033 | Ribosomal protein L29 | 67 | 93 | e-15 | Ribosomal protein L29 [Halorhodospira halophila SL1] | Ribosomal L29 protein |  |
| BgP2658 | Ribosomal protein L18P/L5E | 90 | 100 | e-26 | 50S ribosomal protein L18 [Haemophilus ducreyi 35000HP] | Ribosomal L18p/L5e family |  |
| BgP3870 | 50S ribosomal protein L11 | 143 | 99 | e-60 | ribosomal protein L11 [Hahella chejuensis KCTC 2396] | Ribosomal protein L11, N-terminal domain |  |
| BgP2379 | Ribosomal protein L9 | 150 | 98 | e-40 | 50S ribosomal protein L9 [Pseudomonas putida KT2440] | Ribosomal protein L9, C-terminal domain |  |
| BgP1032 | Ribosomal protein L16 | 137 | 99 | e-59 | 50S ribosomal protein L16 [Xanthomonas campestris] | Ribosomal protein L16 |  |
| BgP4943 | Ribosomal protein S20 | 102 | 85 | e-27 | Ribosomal protein S20p [Pseudomonas fluorescens PfO-1] | Ribosomal protein S20 |  |
| BgP1039 | Ribosomal protein S8 | 130 | 99 | e-41 | 30S ribosomal protein S8 [Vibrio angustum S14] | Ribosomal protein S8 |  |
| BgP3360 | Ribosomal protein L13 | 142 | 97 | e-56 | 50S ribosomal protein L13 [Vibrio angustum S14] | Ribosomal protein L13 |  |
| BgP3056 | Ribosomal protein L20 | 137 | 87 | e-38 | ribosomal protein L20 [Nitrosospira multiformis ATCC 25196] | Ribosomal protein L20 |  |
| BgP2660 | Ribosomal protein L30 | 62 | 95 | e-10 | 50S ribosomal protein L30 [Photorhabdus luminescens] | Ribosomal protein L30p/L7e |  |
| BgP3055 | Ribosomal protein L35 | 64 | 98 | e-10 | ribosomal protein L35 [Methylococcus capsulatus str. Bath] | Ribosomal protein L35 |  |
| BgP1034 | Ribosomal protein S17 | 100 | 80 | e-24 | ribosomal protein S17 [Shewanella denitrificans OS217] | Ribosomal protein S17 |  |
| BgP3939 | Ribosomal protein S15 | 88 | 98 | e-25 | Ribosomal protein S15 [Azotobacter vinelandii AvOP] | Ribosomal protein S15 |  |
| BgP2661 | Ribosomal protein L15 | 151 | 83 | e-35 | 50S ribosomal protein L15 [Oceanospirillum sp. MED92] | Ribosomal protein L15 N-terminal domain |  |
| BgP5475 | Ribosomal protein L3 | 213 | 100 | e-72 | 50S ribosomal protein L3 [Nitrococcus mobilis Nb-231] | Ribosomal protein L3 |  |
| BgP5205 | 30S ribosomal protein S1 | 562 | 95 | <e-200 | 30S ribosomal protein S1 [Nitrococcus mobilis Nb-231] | S1 RNA binding domain |  |
| BgP1038 | Ribosomal protein S14 | 101 | 100 | e-30 | 30S ribosomal protein S14 [Acinetobacter sp. ADP1] | Ribosomal protein S14p/S29e |  |
| BgP1037 | Ribosomal protein L5 | 179 | 99 | e-67 | 50S ribosomal protein L5 [Legionella pneumophila] | ribosomal L5P family C-terminus |  |

Table S4: Ribosomal proteins (PS dataset)
