# Supplementary material for: Insights into the Genome of Large Sulfur Bacteria Revealed by Analysis of Single Filaments
Source: PLoS Biol. 2007 Aug 28;5(9):e230. doi: 10.1371/journal.pbio.0050230 (PMC1951784; doi:10.1371/journal.pbio.0050230)
Supplement: Supplementary file 9 [file pbio.0050230.st005.doc]

| **Orf** | **predicted protein** | **Length (aa)** | **% residues aligned** | **e-value** | **Closest homolog (BLASTx)** | **Pfam domains** |  |
| --- | --- | --- | --- | --- | --- | --- | --- |
| BgP1035 | GOG0093: Ribosomal protein L14 | 122 | 100 | e-44 | ribosomal protein L14 [Nitrococcus mobilis Nb-231] | Ribosomal protein L14p/L23e |  |
| BgP2892 | COG0048: Ribosomal protein S12/S23 | 127 | 97 | e-58 | Ribosomal protein S12 [Nitrosococcus oceani ATCC 19707] | Ribosomal protein S12 |  |
| BgP5474 | COG0088: Ribosomal protein L4/L1e | 218 | 92 | e-51 | 50S ribosomal subunit protein L4 [Erwinia carotovora] | Ribosomal protein L4/L1 family |  |
| BgP5322 | COG0099: Ribosomal protein S13 | 118 | 99 | e-45 | 30S ribosomal protein S13 [Francisella tularensis] | Ribosomal protein S13/S18 |  |
| BgP5320 | COG0522: 30S ribosomal protein S4 | 206 | 100 | e-73 | 30S ribosomal protein S4 [Methylococcus capsulatus str. Bath] | Ribosomal protein S4/S9 N-terminal domain |  |
| BgP1031 | COG0092: 30S ribosomal protein S3 | 211 | 99 | e-91 | ribosomal protein S3 [Alkalilimnicola ehrlichei MLHE-1] | Ribosomal protein S3, C-terminal domain |  |
| BgP4936 | COG0052: Ribosomal protein S2 | 260 | 85 | e-91 | 30S ribosomal protein S2 [Thiomicrospira crunogena XCL-2] | Ribosomal protein S2 |  |
| BgP2891 | COG0049: Ribosomal protein S7 | 156 | 100 | e-54 | Ribosomal protein S7 [Nitrosococcus oceani ATCC 19707] | Ribosomal protein S7p/S5e |  |
| BgP1029 | COG0185: Ribosomal protein S19 | 92 | 100 | e-36 | 30S ribosomal protein S19 [Vibrio splendidus 12B01] | Ribosomal protein S19 | |
| BgP1030 | COG0091: Ribosomal protein L22 | 109 | 100 | e-38 | Ribosomal protein L22 [Shewanella sp. PV-4] | Ribosomal protein L22p/L17e |  |
| BgP5321 | COG0100: Ribosomal protein S11 | 129 | 100 | e-55 | 30S ribosomal subunit protein S11 [Escherichia coli K12] | Ribosomal protein S11 |  |
| BgP2659 | COG0098: Ribosomal protein S5 | 170 | 99 | e-53 | 30S ribosomal protein S5 [Congregibacter litoralis KT71] | Ribosomal protein S5, N-terminal domain |  |
| BgP5475 | COG0087: Ribosomal protein L3 | 213 | 100 | e-72 | 50S ribosomal protein L3 [Nitrococcus mobilis Nb-231] | Ribosomal protein L3 |  |
| Bg1028 | COG0090: Ribosomal protein L2 | 224 | 100 | e-93 | Single-strand binding protein [Saccharophagus degradans] | Ribosomal Proteins L2, C-terminal domain |  |
| BgP3869 | COG0081: Ribosomal protein L1 | 201 | 100 | e-72 | 50S ribosomal protein L1 [Methylococcus capsulatus str. Bath] | Ribosomal protein L1p/L10e family |  |
| BgP2658 | COG0256: Ribosomal protein L18P/L5E | 90 | 100 | e-26 | 50S ribosomal protein L18 [Haemophilus ducreyi 35000HP] | Ribosomal L18p/L5e family |  |
| BgP3870 | COG0080: 50S ribosomal protein L11 | 143 | 99 | e-60 | ribosomal protein L11 [Hahella chejuensis KCTC 2396] | Ribosomal protein L11, N-terminal domain |  |
| BgP1032 | COG0197: Ribosomal protein L16 | 137 | 99 | e-59 | 50S ribosomal protein L16 [Xanthomonas campestris] | Ribosomal protein L16 |  |
| BgP1039 | COG0096: Ribosomal protein S8 | 130 | 99 | e-41 | 30S ribosomal protein S8 [Vibrio angustum S14] | Ribosomal protein S8 |  |
| BgP3360 | COG0102: Ribosomal protein L13 | 142 | 97 | e-56 | 50S ribosomal protein L13 [Vibrio angustum S14] | Ribosomal protein L13 |  |
| BgP1034 | COG0186: Ribosomal protein S17 | 100 | 80 | e-24 | ribosomal protein S17 [Shewanella denitrificans OS217] | Ribosomal protein S17 |  |
| BgP3939 | COG0184: Ribosomal protein S15 | 88 | 98 | e-25 | Ribosomal protein S15 [Azotobacter vinelandii AvOP] | Ribosomal protein S15 |  |
| BgP2661 | COG0200: Ribosomal protein L15 | 151 | 83 | e-35 | 50S ribosomal protein L15 [Oceanospirillum sp. MED92] | Ribosomal protein L15 N-terminal domain |  |
| BgP1037 | COG0094: Ribosomal protein L5 | 179 | 99 | e-67 | 50S ribosomal protein L5 [Legionella pneumophila] | ribosomal L5P family C-terminus |  |
| BgP5130 | COG0085: RNA polymerase beta SU | 1118 | 100 | <e-200 | DNA-directed RNA polymerase, beta subunit [Nitrosococcus oceani] | RNA polymerase Rpb2, domain 6 |  |
| BgP2764 | COG0016: Phenylalanyl-tRNA synthetase | 174 | 94 | e-42 | Phenylalanyl-tRNA synthetase, beta subunit [Pseduomonas fluorescens] |  |  |
| BgP0791** | COG0016: Phenylalanyl-tRNA synthetase | 266 | 99 | e-120 | Phenylalanyl-tRNA synthetase, beta subunit [Methylococcus capsulatus str. Bath] | Ferredoxin-fold anticodon binding domain |  |
| BgP0832* | COG0525: Valyl-tRNA synthetase | 236 | 69 | e-47 | Valyl-tRNA synthetase [Thiomicrospira crunogena XCL-2] | Anticodon binding domain |  |
| BgP0833* | COG0525: Valyl-tRNA synthetase | 138 | 100 | e-31 | Valyl-tRNA synthetase [Alkalilimnicola ehrlichei MlHe-1] |  |  |
| BgP6502** | COG0525: Valyl-tRNA synthetase | 100 | 97 | e-39 | Valyl-tRNA synthetase, beta subunit [Methylococcus capsulatus] |  |  |
| BgP5158* | COG0124: Histidyl-tRNA synthetase | 140 | 90 | e-33 | Histidyl-tRNA synthetase [Hahella chejuensis KCTC2396] |  |  |
| BgP5159* | COG0124: Histidyl-tRNA synthetase | 345 | 94 | e-107 | Histidyl-tRNA synthetase class IIa [Thiomicrospira crunogena XCL-2] | Anticodon binding domain |  |
| BgP2968 | COG0495: Leucyl-tRNA synthetase | 622 | 97 | <e-200 | Leucyl-tRNA synthetase class Ia [Nitrosococcus oceani] | Anticodon binding domain |  |
| BgP0391 | COG0201: SecY protein | 270 | 99 | e-100 | SecY protein [Nitrosococcus oceani] | secY protein |  |
|  |  |  |  |  |  |  |  |

Table S5: Single copy genes I (PS dataset)
