# Supplementary material for: Insights into the Genome of Large Sulfur Bacteria Revealed by Analysis of Single Filaments
Source: PLoS Biol. 2007 Aug 28;5(9):e230. doi: 10.1371/journal.pbio.0050230 (PMC1951784; doi:10.1371/journal.pbio.0050230)
Supplement: Supplementary file 10 [file pbio.0050230.st006.doc]

Table S6: Single copy genes II (PS dataset)

| **Orf** | **Length (aa)** | **% residues**  **aligned** | **e-value** | **Predicted protein/closest homolog (BLASTx)** | **Pfam domains** |
| --- | --- | --- | --- | --- | --- |
| BgP0013 | 197 | 100 | e-88 | Imidazoleglycerol-phosphate dehydratase [Hahella chejuensis KCTC 2396] | Imidazoleglycerol-phosphate dehydratase |
| BgP0761 | 95 | 100 | e-27 | glutamyl-tRNA(Gln) amidotransferase, C subunit [Alkalilimnicola ehrlichei MLHE-1] | Glu-tRNAGln amidotransferase C subunit |
| BgP0992 | 183 | 96 | e-57 | elongation factor Ts [Methylococcus capsulatus str. Bath] | Elongation factor TS |
| BgP1031 | 211 | 99 | e-91 | ribosomal protein S3 [Alkalilimnicola ehrlichei MLHE-1] | Ribosomal protein S3, C-terminal domain |
| BgP1032 | 137 | 99 | e-59 | 50S ribosomal protein L16 [Xanthomonas campestris pv. campestris str. ATCC 33913] | Ribosomal protein L16 |
| BgP1033 | 67 | 93 | e-15 | Ribosomal protein L29 [Halorhodospira halophila SL1] | Ribosomal L29 protein |
| BgP1057 | 125 | 91 | e-41 | 30S ribosomal protein S6 [Vibrio angustum S14] | Ribosomal protein S6 |
| BgP1088 | 357 | 98 | e-103 | Glycine--tRNA ligase [Alkalilimnicola ehrlichei MLHE-1] | Glycyl-tRNA synthetase beta subunit |
| BgP1594 | 152 | 95 | e-38 | D-tyrosyl-tRNA(Tyr) deacylase [Pseudomonas fluorescens PfO-1] | D-Tyr-tRNA(Tyr) deacylase |
| BgP1696 | 394 | 99 | e-79 | Trigger factor [Azotobacter vinelandii AvOP] | Bacterial trigger factor protein (TF) |
| BgP1775 | 71 | 86 | e-17 | RNA polymerase, omega subunit [marine gamma proteobacterium HTCC2207] | RNA polymerase Rpb6 |
| BgP2198 | 172 | 96 | e-19 | hypothetical protein RED65_01155 [Oceanobacter sp. RED65] | Uncharacterized ACR, COG1399 |
| BgP2379 | 150 | 98 | e-40 | 50S ribosomal protein L9 [Pseudomonas putida KT2440] | Ribosomal protein L9, C-terminal domain |
| BgP2379 | 150 | 98 | e-40 | 50S ribosomal protein L9 [Pseudomonas putida KT2440] | Ribosomal protein L9, C-terminal domain |
| BgP2661 | 151 | 83 | e-35 | 50S ribosomal protein L15 [Oceanospirillum sp. MED92] | Ribosomal protein L15 amino terminus |
| BgP2764 | 174 | 94 | e-42 | phenylalanyl-tRNA synthetase, beta subunit [Pseudomonas fluorescens Pf-5] | Ferredoxin-fold anticodon binding domain |
| BgP2841 | 135 | 98 | e-08 | Protein-export membrane protein [Alcanivorax borkumensis SK2] | Preprotein translocase SecG subunit |
| BgP3017 | 200 | 44 | e-27 | ribosomal protein S16 [Alkalilimnicola ehrlichei MLHE-1] | Ribosomal protein S16 |
| BgP3056 | 137 | 87 | e-38 | ribosomal protein L20 [Nitrosospira multiformis ATCC 25196] | Ribosomal protein L20 |
| BgP3103 | 122 | 96 | e-42 | ribosomal protein L19 [Vibrio cholerae O1 biovar eltor str. N16961] | Ribosomal protein L19 |
| BgP3105 | 154 | 100 | e-53 | tRNA (guanine-N(1)-)-methyltransferase [Oceanospirillum sp. MED92] | tRNA (Guanine-1)-methyltransferase |
| BgP3106 | 168 | 98 | e-46 | 16S rRNA processing protein RimM [Nitrosococcus oceani ATCC 19707] | PRC-barrel domain |
| BgP3361 | 115 | 100 | e-25 | Iojap-related protein [Chromohalobacter salexigens DSM 3043] | Domain of unknown function DUF143 |
| BgP3369 | 348 | 98 | e-90 | Negative regulator of class I heat shock protein [Nitrosococcus oceani ATCC 19707] | HrcA protein C terminal domain |
| BgP3490 | 116 | 60 | e-25 | Ribosomal protein L27 [Marinobacter aquaeolei VT8] | Ribosomal L27 protein |
| BgP3491 | 184 | 54 | e-17 | 50S ribosomal protein L21 [Alcanivorax borkumensis SK2] | Ribosomal prokaryotic L21 protein |
| BgP3872 | 187 | 99 | e-71 | transcription antitermination protein NusG [Halorhodospira halophila SL1] | Transcription termination factor nusG |
| BgP3873 | 126 | 85 | e-17 | preprotein translocase, SecE subunit [Shewanella sp. MR-7] | SecE/Sec61-gamma subunits of protein |
| BgP3941 | 122 | 98 | e-22 | ribosome-binding factor A [Pseudomonas putida KT2440] | Ribosome-binding factor A |
| BgP3942 | 747 | 94 | <e-200 | hypothetical protein CburR_01001431 [Coxiella burnetii RSA 331] | Elongation factor Tu GTP binding domain |
| BgP4924 | 124 | 100 | e-35 | Ribosomal protein L7/L12 [Nitrosococcus oceani ATCC 19707] | Ribosomal protein L7/L12 C-terminus |
| BgP4937 | 181 | 95 | e-36 | COG0319: Predicted metal-dependent hydrolase [Haemophilus somnus 2336] | Uncharacterized protein family UPF005 |
| BgP4943 | 102 | 85 | e-27 | Ribosomal protein S20p [Pseudomonas fluorescens PfO-1] | Ribosomal protein S20 |
| BgP5097 | 129 | 87 | e-20 | ribonuclease P protein component [Erwinia carotovora subsp. atroseptica SCRI1043] | Ribonuclease P |
| BgP5223 | 144 | 97 | e-42 | Protein of unknown function DUF150 [Azotobacter vinelandii AvOP] | Uncharacterised BCR, YhbC family COG07 |
| BgP5676 | 322 | 89 | e-105 | Signal recognition particle GTPase [Idiomarina loihiensis L2TR] | SRP54-type protein, GTPase domain |
| BgP5803 | 155 | 96 | e-43 | Holliday junction resolvase [Vibrio vulnificus YJ016] | Crossover junction endodeoxyribonuclease |
| BgP5854 | 161 | 96 | e-51 | MECDP-synthase [Clostridium thermocellum ATCC 27405] | YgbB family |
| BgP6683 | 59 | 85 | e-12 | Holliday junction DNA helicase RuvB [Alteromonas macleodii 'Deep ecotype'] | Holliday junction DNA helicase ruvB N-terminus |
|  |  |  |  |  |  |
