# Supplementary material for: Insights into the Genome of Large Sulfur Bacteria Revealed by Analysis of Single Filaments
Source: PLoS Biol. 2007 Aug 28;5(9):e230. doi: 10.1371/journal.pbio.0050230 (PMC1951784; doi:10.1371/journal.pbio.0050230)
Supplement: Supplementary file 11 [file pbio.0050230.st007.doc]

| **Orf** | **Length (aa)** | **% residues**  **aligned** | **e-value** | **Predicted protein/closest homolog (BLASTx)** | **Pfam domains** |
| --- | --- | --- | --- | --- | --- |
| BgP1383 | 357 | 100 | e-105 | Alanyl-tRNA synthetase [Nitrosococcus oceani ATCC 19707] | DHHA1 domain |
| BgP4065 | 585 | 97 | <e-200 | Arginyl-tRNA synthetase, class Ic [Alkalilimnicola ehrlichei MLHe-1] | tRNA synthetases class I (R) |
| BgP5189 | 595 | 98 | <e-200 | Aspartyl-tRNA synthetase [Alkalilimnicola ehrlichei MLHe-1] | tRNA synthetases class II (D, K and N) |
| BgP0669 | 463 | 100 | e-170 | Cysteinyl-tRNA synthetase, class Ia [Methylococcus capsulatus str. Bath] | tRNA synthetases class I (C) catalyti |
| BgP2523 | 467 | 98 | e-175 | Glutamyl-tRNA synthetase [Alkalilimnicola ehrlichei MLHe-1] | tRNA synthetases class I (E and Q) |
| BgP6047 | 551 | 97 | <e-200 | Glutaminyl-tRNA synthetase, class Ic [Syntrophobacter fumaroxidans] | tRNA synthetases class I (E and Q) |
| BgP2090 | 296 | 98 | e-136 | Glycyl-tRNA synthetase, alpha chain [Hahella chejuensis KCTC 2396] | Glycyl-tRNA synthetase alpha subunit |
| BgP1088 | 357 | 98 | e-103 | Glycyl-tRNA synthetase, beta chain [Alkalilimnicola ehrlichei MLHe-1] | Glycyl-tRNA synthetase beta subunit |
| BgP5158* | 140 | 90 | e-33 | Histidyl-tRNA synthetase [Hahella chejuensis KCTC 2396] |  |
| BgP5159* | 345 | 94 | e-107 | Histidyl-tRNA synthetase, class IIa [Thiomicrospira crunogena XCL-2] | Anticodon binding domain |
| BgP1369* | 212 | 100 | e-71 | Isoleucyl-tRNA synthetase, [Nitrosococcus oceani ATCC 19707] | Anticodon-binding domain |
| BgP1371* | 638 | 100 | <e-200 | Isoleucyl-tRNA synthetase, class Ia [Methylococcus capsulatus str. Bath] | tRNA synthetases class I (I, L, M and |
| BgP1370* | 134 | 70 | e-33 | Isoleucyl-tRNA synthetase, class Ia [Nitrococcus mobilis Nb-231] |  |
| BgP2968 | 622 | 97 | <e-200 | Leucyl-tRNA synthetase, class Ia [Nitrosococcus oceani ATCC 19707] | Anticodon-binding domain |
| BgP5948 | 227 | 97 | e-20 | Lysyl-tRNA synthetase [Wolbachia endosymbiont] |  |
| BgP4948** | 185 | 97 | e-45 | Lysyl-tRNA synthetase [Nitrococcus mobilis Nb-231] | tRNA synthetases class II (D, K and N) |
| BgP0633 | 273 | 99 | e-83 | Methionyl-tRNA synthetase [Oceanospirillum sp. MED92] | Putative tRNA binding domain |
| BgP0634* | 398 | 99 | e-179 | Methionyl-tRNA synthetase [Nitrococcus mobilis Nb-231] |  |
| BgP2764 | 174 | 94 | e-42 | Phenylalanyl-tRNA synthetase, beta subunit [Pseudomonas fluorescens] | Ferredoxin-fold anticodon binding dom |
| BgP0791** | 266 | 99 | e-120 | Phenylalanyl-tRNA synthetase, class IIc [Methylococcus capsulatus] | tRNA synthetases class II core domain |
| BgP0653 | 183 | 99 | e-63 | Prolyl-tRNA synthetase [Shewanella amazonensis SB2B] | Anticodon binding domain |
| BgP5571 | 390 | 99 | e-171 | Threonyl-tRNA synthetase, class IIa [Alkalilimnicola ehrlichei MLHe-1] | tRNA synthetase class II core domain |
| BgP5570* | 191 | 84 | e-55 | Threonyl-tRNA synthetase, class IIa [Nitrosococcus oceani ATCC 19707] | TGS domain |
| BgP5796 | 403 | 98 | e-161 | Tryptophanyl-tRNA synthetase [Nitrosococcus oceani ATCC 19707] | tRNA synthetases class I (W and Y) |
| BgP4903 | 400 | 100 | e-157 | Tyrosyl-tRNA synthetase, class Ib [Alkalilimnicola ehrlichei MLHe-1] | tRNA synthetases class I (W and Y) |
| BgP0832* | 236 | 69 | e-47 | Valyl-tRNA Synthetase [Thiomicrospira crunogena XCL-2] | Anticodon-binding domain |
| BgP0833* | 138 | 100 | e-31 | Valyl-tRNA Synthetase [Alkalilimnicola ehrlichei MLHe-1] |  |
| BgP6502** | 100 | 97 | e-39 | Valyl-tRNA Synthetase [Methylococcus capsulatus str. Bath] |  |

Table S7: tRNA synthetases (PS dataset)
