# Supplementary material for: Insights into the Genome of Large Sulfur Bacteria Revealed by Analysis of Single Filaments
Source: PLoS Biol. 2007 Aug 28;5(9):e230. doi: 10.1371/journal.pbio.0050230 (PMC1951784; doi:10.1371/journal.pbio.0050230)
Supplement: Supplementary file 12 [file pbio.0050230.st008.doc]

| **Orf** | **Length (aa)** | **% residues**  **aligned** | **e-value** | **Predicted protein** | **Closest homolog (BLASTx)** | **% identity/**  **similarity** |
| --- | --- | --- | --- | --- | --- | --- |
| BgP1671 | 947 | 92 | e-73 | Large exoprotein containing haemagglutination activity domain | filamentous haemagglutinin family outer membrane protein [Trichodesmium erythraeum IMS101] | 27/44 |
| BgP3606 | 876 | 91 | e-48 | Large exoprotein containing haemagglutination activity domain | COG3210: Large exoproteins involved in heme utilization or adhesion [Nostoc punctiforme PCC 73102] | 26/41 |
| BgP5222 | 851 | 93 | e-73 | Large protein containing haemagglutination activity domain | COG3210: Large exoproteins involved in heme utilization or adhesion [Nostoc punctiforme PCC 73102] | 28/46 |
| BgP3860 | 850 | 90 | e-69 | Large exoprotein containing haemagglutination activity domain | conserved hypothetical proteinall5113 [Nostoc sp. PCC 7120] | 26/43 |
| BgP2037 | 813 | 69 | e-59 | Large exoprotein containing haemagglutination activity domain | conserved hypothetical protein alr1906 [Nostoc sp. PCC 7120] | 30/47 |
| BgP1576 | 736 | 92 | e-58 | Large exoprotein containing haemagglutination activity domain | COG3210: Large exoproteins involved in heme utilization or adhesion [Nostoc punctiforme PCC 73102] | 27/43 |
| BgP3617 | 648 | 88 | e-47 | Large protein containing haemagglutination activity domain | Filamentous haemagglutinin-like [Anabaena variabilis ATCC 29413] | 26/42 |
| BgP0050 | 442 | 60 | e-23 | Large exoprotein containing haemagglutination activity domain | filamentous haemagglutinin family outer membrane protein [Trichodesmium erythraeum IMS101] | 32/47 |
| BgP1511 | 239 | 91 | e-28 | Large exoprotein containing haemagglutination activity domain | Filamentous haemagglutinin-like [Anabaena variabilis ATCC 29413] | 33/52 |
| BgP3497 | 222 | 80 | e-27 | large exoprotein containing haemagglutination activity domain | Filamentous haemagglutinin-like [Anabaena variabilis ATCC 29413] | 37/55 |
| BgP0248 | 201 | 84 | e-26 | Large exoprotein containing haemagglutination activity domain | conserved hypothetical protein all7313 [Nostoc sp. PCC 7120] | 44/60 |
| BgP1153 | 162 | 83 | e-23 | Large exoprotein containing haemagglutination activity domain | conserved hypothetical protein all7313 [Nostoc sp. PCC 7120] | 44/65 |

Table S8: Haemagglutinin-domain containing proteins (PS dataset)
